# Translation and evaluation of a pre-clinical 5-protein response prediction signature in a breast cancer phase Ib clinical trial

**DOI:** 10.1371/journal.pone.0213892

**Published:** 2019-03-21

**Authors:** Axel Ducret, Ian James, Sabine Wilson, Martina Feilke, Andreas Tebbe, Nikolaj Dybowski, Sarah Elschenbroich, Martin Klammer, Adele Blackler, Wei-Li Liao, Yuan Tian, Thomas Friess, Birgit Bossenmaier, Gabriele Dietmann, Christoph Schaab, Todd Hembrough, Maurizio Ceppi

**Affiliations:** 1 Roche Pharmaceutical Research and Early Development, Roche Innovation Center Basel, Basel, Switzerland; 2 A4P Consulting Ltd, Sandwich, United Kingdom; 3 Roche Pharmaceutical Research and Early Development, Roche Innovation Center Munich, Penzberg, Germany; 4 Evotec (München) GmbH, Munich, Germany; 5 Oncoplex Diagnostics, Rockville, MD, United States of America; Fondazione IRCCS Istituto Nazionale dei Tumori, ITALY

## Abstract

Human protein biomarker discovery relies heavily on pre-clinical models, in particular established cell lines and patient-derived xenografts, but confirmation studies in primary tissue are essential to demonstrate clinical relevance. We describe in this study the process that was followed to clinically translate a 5-protein response signature predictive for the activity of an anti-HER3 monoclonal antibody (lumretuzumab) originally measured in fresh frozen xenograft tissue. We detail the development, qualification, and validation of the multiplexed targeted mass spectrometry assay used to assess the signature performance in formalin-fixed, paraffin-embedded human clinical samples collected in a phase Ib trial designed to evaluate lumretuzumab in patients with metastatic breast cancer. We believe that the strategy delineated here provides a path forward to avoid the time- and cost-consuming step of having to develop immunological reagents against unproven targets. We expect that mass spectrometry-based platforms may become part of a rational process to rapidly test and qualify large number of candidate biomarkers to identify the few that stand a chance for further development and validation.

## Introduction

In the last few years, pre-clinical research has gradually shifted from studying cell lines to patient-derived xenografts (PDX) as source of biomarkers, in particular in the oncology arena [1–3]. In breast cancer, for instance, a consortium of academic laboratories from Europe, Australia, and North America has recently described and released data on over 500 stably transplantable PDX models representing all three clinical subtypes of breast cancer (reviewed in detail in [2]) with most of these models characterized with respect to their genetics, transcriptomics and proteomics features. Remarkably, PDX models have been shown to retain a significant degree of biological and histological fidelity with their tumors of origin. Thus, a recent study including 22 patient-derived breast cancer xenografts demonstrated that PDX tumors recapitulated the proteomic diversity of human breast cancers [4], and, more specifically, that the proteogenomic signatures of PDXs resembled most findings from breast cancer patients.

An attractive aspect of PDX models is that they may show comparable responses to the originating tumor, which make them suitable for screening against specific therapeutics [2] and for discovering biomarkers for drug sensitivity and response. Lumretuzumab (RG7116) is a humanized, glycoengineered immunoglobulin-G1 antibody that binds with high sensitivity and specificity to the extracellular domain of the human epidermal growth factor receptor 3 (HER3) [5], one of the members of HER family receptors playing a critical role in tumor growth, proliferation, and progression of numerous epithelial malignancies (reviewed in [6,7]). In particular, HER3 up-regulation has been correlated with resistance to HER2-targeting inhibitors in breast cancer due to the unique ability of the HER2:HER3 heterodimer to activate the PI3K/AKT-mTOR signaling pathway (reviewed in [8–10]). Lumretuzumab hinders the binding of heregulin (a native HER3 ligand) to HER3, resulting in almost complete inhibition of HER3 heterodimerization and subsequent phosphorylation, and causing tumor arrest of cell line–based xenografts in mouse models up to complete remission compared to controls. A first in-human, dose escalation phase I study to characterize safety, efficacy, and pharmacokinetic and pharmacodynamic properties of lumretuzumab recently reported that the molecule was well tolerated and showed evidence of clinical activity [11]. However, the roles of HER3 or heregulin as prognostic markers for HER3-targeted treatment response have remained controversial and no marker have been identified so far for breast cancer. High heregulin expression in association with activated HER3 has been proposed as an actionable biomarker in patients with squamous cell carcinomas of the head and neck [12] but the markers failed to show promise in a clinical trial setting [13,14]. Moreover, in a recent study, a combined lumretuzumab plus erlotinib treatment in the squamous non-small-cell lung carcinoma extension cohort showed no evidence of meaningful clinical benefit despite enriching for tumors with higher heregulin mRNA expression levels [15]. Importantly, heregulin mRNA expression is generally low in breast cancer ([15], as also observed in the TCGA dataset) and it may therefore not be usable as a prognostic marker.

In a complementary approach, we recently identified a 5-protein signature predictive for lumretuzumab activity in cell line- and patients-derived xenograft tumors using a global proteomics strategy, sparking great interest whether the signature could be assessed and potentially confirmed in a breast cancer clinical trial. However, the signature needed to be translated from a fresh-frozen xenograft tissue (where it was discovered) to formalin-fixed tumor tissue (the material collected in the clinical trial) under very tight timelines, requiring a well devised strategy and careful considerations of methods and potential risks with respect to prevalence and modulation of the proteins of interest in the clinical samples.

In this study, we describe the development, qualification, and validation of a multiplexed targeted mass spectrometric (MS) assay to measure a response prediction protein signature in human clinical samples collected in a phase Ib trial (https://clinicaltrials.gov/ct2/show/NCT01918254; [16]). We first demonstrated that the 5 proteins of interest were measurable in breast cancer tissue after which we re-developed and qualified the MS assays for the samples collected in the clinical trial. We then investigated whether the protein signature correlated with the actual tumor response to lumretuzumab treatment according to RECIST criteria.

## Materials and methods

### Cell line- and patient tumor-derived xenograft analysis using discovery and targeted mass spectrometry

The discovery proteomics effort described here is performed similarly to the strategy described in Geiger *et al*. [17] except that a protein fractionation step using 10% NuPAGE MES SDS-PAGE was used instead of a peptide-based fractionation by strong-cation exchanger chromatography. Briefly, responder/non-responder human xenografts obtained from cell lines or patient-derived tumor fragments (S1 Table) were lysed by sonication in 8 M urea, 1% (w/v) CHAPS, 50 mM Tris-Cl pH 8.8 in presence of protease inhibitors (Complete, Roche). 50 μg of protein lysates were mixed 1:1 with a mix of SILAC-labelled reference cell lines and subjected to SDS-PAGE analysis. The gel was divided in 12 horizontal bands (based on the mass molecular marker) and proteins from each individual sample were subjected to in-gel trypsin digestion. Approximately 1–2 μg of each sample were analyzed in duplicate using a data-dependent acquisition strategy by nano liquid chromatography electrospray tandem mass spectrometry using a Proxeon Biosystems HPLC coupled to a LTQ-Orbitrap Velos tandem mass spectrometer (Thermo Fisher, Waltham, MA). Peptide separation was achieved with a home-packed 75 μm i.d. column packed with 1.8 μm Reprosil C_18_ A.Q. reverse phase material (Dr. Maisch)) using 130 min gradients at a flow rate of 200 nL/min. The mass spectrometer was operated in a data-dependent acquisition mode to automatically switch between full scans in the Orbitrap mass analyzer (resolution = 60,000) and the acquisition of CID fragmentation spectra (top N = 15, with dynamic exclusion enabled) in the linear ion trap (LTQ). Protein identification and relative quantification to the internal SILAC-labelled proteins was performed essentially as described in [17] using MaxQuant version 1.2.0.11 [18] against the Uniprot protein database release 12_2010 (filtered for “human”, 76767 entries; http://www.uniprot.org). Data were searched with a mass tolerance of 20 ppm for parent ions and 0.5 Th for fragment ions with methionines (reduced/oxidized; +15.9949 Da), lysines (labelled: +8.0142 Da) and arginines (labelled: +10.00827 Da) considered as differential modifications and cysteines considered as fully carbamidomethylated (+57.0199 Da). Peptides and proteins identifications were filtered at a False Discovery Rate (FDR) ≤1%.

Targeted quantitative analysis of the 5 putative protein candidates was performed using the same protein lysates as described above. A total of 0.5–2 μg protein digest was analyzed in triplicate on a HPLC system using a 75 μm inner diameter x 15 cm length chromatographic column packed with C18 resin (ProntoSIL 200-5-C18AQ; Bischoff Chromatography, Germany) coupled to a triple quadrupole mass spectrometer (TSQ Vantage Quantum, Thermo Scientific, Waltham, MA). The mass spectrometer was operated in SRM mode using a total cycle time of 1 s and the two quadrupoles 1 and 3 were set at a width of 0.7 Th (S2 Table). For protein quantitation, peak areas from each endogenous and spiked internal heavy label standard peptide (typically, 50 fmol of AQUA grade peptide; Thermo Scientific, Waltham, MA) were calculated and ratios were determined using Skyline version 1.3 (https://skyline.ms/project/home/software/Skyline/begin.view). SRM assays were qualified by generating reversed standard curves for the 17 peptides belonging to the 5 proteins of interest to define assay limit of detection (LOD) and lower limit of quantification (LLOQ). For each analyte, 1.5 amol—100 fmol (12 dilution points) of the synthetic heavy peptide were spiked into a pooled digest of the xenograft matrix containing 50 fmol of the light label peptide. LOD was determined by identifying the lowest concentration in the standard curve for which relative error and accuracy were lower than 25%. LLOQ was set as 3-fold LOD. Quantitation of the endogenous analytes was determined using the median of all measured transitions.

### Tumor biopsy samples

The SRM method development and qualification was performed in commercially acquired Breast Cancer and Non Small Cell Lung Cancer human tissue samples and the xenograft models (formalin-fixed) mentioned in the previous section.

The tumor biopsy samples investigated in this study were obtained from patients with HER3-positive, HER2-non-amplified metastatic breast cancer enrolled in the clinical trial BP27852, an open-label, multicenter, dose-escalation study to evaluate the safety, pharmacokinetics and activity of lumretuzumab (https://clinicaltrials.gov/ct2/show/NCT01918254; [16]). Local ethics committee approval was obtained and all patients provided written informed consent. The study was conducted in accordance with Good Clinical Practice guidelines and the Declaration of Helsinki in nine centers in Denmark, France, Germany and Spain. The following ethics committees approved the study: Denmark (Videnskabsetiske Komiteer Region Hovedstaden on 24 Apr 2013), Spain (Hospital Clinico Universitario de Valencia CEIC on 07 May 2013; CEIC Hospital Universitario 12 de Octubre on 07 May 2013; CEIC Parc de Salut Mar; IMIM- Hospital del Mar, C/ Dr. Aiguader, 88, Planta 1, 08003, Barcelona on 07 May 2013; CEIC Hospital Vall D’Hebron on 15 Oct 2014), Germany (Ethikkommission der Medizinischen Fakultät Heidelberg on 21 Aug 2013 and on 23 Feb 2015) and France (Comité de protection des personnes Ile de France III on 17 Jun 2013 and on 30 Jun 2013). Eligible patients were dosed with lumretuzumab in combination with pertuzumab and paclitaxel and clinical response was assessed using RECIST 1.1 criteria [19]. Residual material from pre-dose FFPE tumor biopsy samples was made available from 32 patients. IHC assays for EGFR, HER2, and HER3 were performed and analyzed as described previously [11].

### Targeted mass spectrometry assay development and qualification

Fit-for-purpose assay development and qualification for the 5 proteins constituting the response prediction signature was performed essentially as previously described [20]. Briefly, commercially available purified recombinant proteins were digested with trypsin in Liquid Tissue buffer (Oncoplex Dx) and unique peptides from each protein were monitored using Selected Reaction Monitoring (SRM). Cell lines were first fixed in formalin and embedded in paraffin prior to sample processing. To analyze FFPE tumor tissues, one 5 μm section was placed on glass slide for hematoxylin and eosin staining, to guide tumor area selection, while one to several consecutive 10 μm sections (stained with hematoxylin) were placed on Director slides and laser microdissected (Molecular Machines & Industries, Eching, Germany). Collected tumor tissue was solubilized and digested with trypsin using Liquid Tissue according to manufacturer’s instructions. Total peptide concentration from each sample was measured by a micro bicinchoninic acid assay (Thermo Fisher Scientific Inc, Waltham, MA).

A total of 1 μg protein digest was analyzed in triplicate on a nanoACQUITY liquid chromatography system (Waters Corporation, Milford, MA) using a 100 μm inner diameter x 15 cm length chromatographic column packed with C18 resin (ProntoSIL 200-5-C18AQ; Bischoff Chromatography, Germany) coupled to a triple quadrupole mass spectrometer (TSQ Quantiva, Thermo Scientific, Waltham, MA). The mass spectrometer was operated in SRM mode using a total cycle time of 1 s and the two quadrupoles 1 and 3 were set at a width of 0.7 Th (S3 Table). For protein quantitation, peak areas from each endogenous and spiked internal heavy label standard peptide (typically, 5 fmol of AQUA grade peptide; Thermo Scientific, Waltham, MA) were calculated and ratios were determined using PinPoint 1.3 (Thermo Scientific, Waltham, MA).

SRM assays were qualified by generating standard curves for the 14 peptides belonging to the 5 proteins of interest to define assay limit of detection (LOD) and lower limit of quantification (LLOQ). For each analyte, 25 amol—25 fmol (14 dilution points) of the synthetic light peptide were spiked into a *P*. *furiosus* protein matrix (Agilent Technologies Inc., Santa Clara, CA) containing 5 fmol of the heavy label peptide. LOD was determined by identifying the lowest concentration in the standard curve for which coefficient of variation (CV) and accuracy were lower than 20%, respectively better than 80%. LLOQ was determined by identifying the next highest concentration of the standard curve above the LOD. Quantitation of the endogenous analyte was determined using all available product ions.

SRM measurements for EGFR, HER2, and HER3 were performed concomitantly as described previously [20].

### Biostatistics

Missing values for peptides that were measured below LLOQ (as determined by SRM) were imputed using the following procedure. Peptides measured with a concentration below LLOQ were assigned a random concentration drawn from a uniform distribution over a range from 0 to LLOQ amol/μg lysate. If more than one peptide value from the same protein was missing per measurement, an additional draw was then performed from a normal distribution, using the previously drawn random number as mean and a CV of 25%. This step was used to generate a variation assumed to be realistic for data at such low concentrations.

All plots presented in this study have been generated using the JMP program version 10.0.1 (SAS, NC, USA; http://www.jmp.com).

## Results

### Discovery and qualification of a response prediction signature to lumretuzumab in pre-clinical models

**Fig 1:**
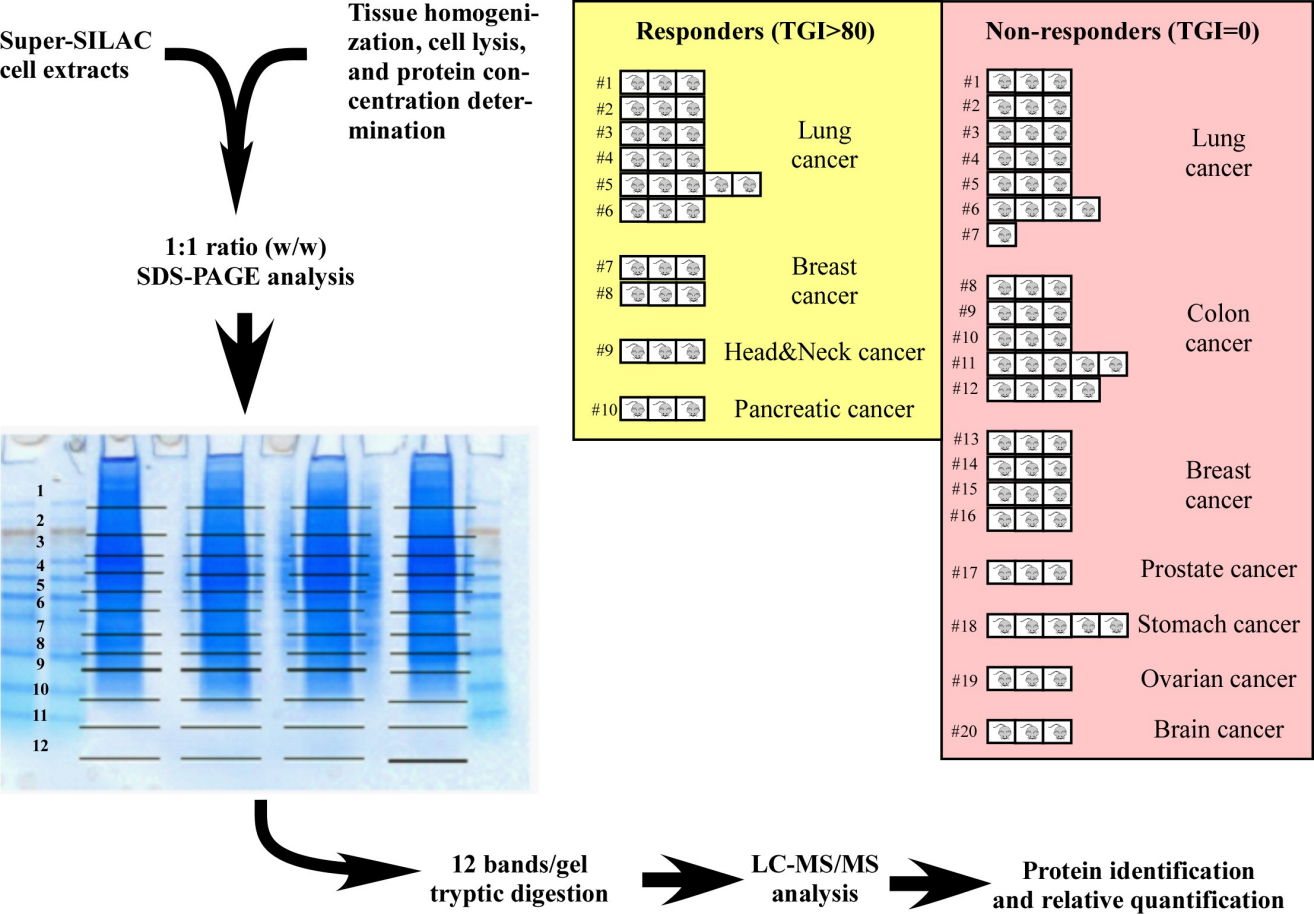
Proteomics strategy leading to the discovery of the 5-protein signature predictive to lumretuzumab activity. Cell- or patient-derived xenografts responsive (TGI>80) or resistant to lumretuzumab activity were lysed and mixed 1:1 (w/w) with super-SILAC-labelled cell extracts [17] prior to SDS-PAGE fractionation, tryptic digestion, and LC-MS/MS analysis. Protein identification and quantification relative to the super-SILAC standards was performed as previously described [17]. The lineage and the number of different cell- and patient-derived xenograft models included in the discovery step are indicated, as is the number of replicates per xenograft samples.

A large-scale unbiased proteomics discovery strategy (Fig 1) was conducted to identify a protein biomarker or a protein signature predictive for response to lumretuzumab from a set of 30 cell line- and patient-derived xenografts (10 responders and 20 non-responders) of diverse lineages. Data normalization was performed using the super-SILAC relative quantification method [17] resulting in the identification of approximately 4000 different protein groups with overall 98% non-missing data points after median summarization (on average 3000 proteins identified per xenograft model with 96% non-missing data points).

In a first approach, a univariate data analysis using a mixed linear model (on individual replicates) or the Wilcoxon test (on median) was performed considering only protein groups with at least 75% of non-missing values per group. 12 proteins fulfilled a (predefined) absolute effect size criterion of >3-fold but without reaching significance (predefined as <0.05 FDR). In a second step, a response prediction signature was established using a support vector machine-based classifier. Feature selection was performed to select a sparse protein signature reaching predefined criteria of 95% sensitivity and 90% specificity. The resulting 5-feature signature (DPYSL2: dihydropyrimidinase-related protein 2; OAT: ornithine aminotransferase, mitochondrial; CLIC3: chloride intracellular channel protein 3; GM2A: ganglioside GM2 activator; PADI3: peptidylarginine deiminase III) fulfilled the pre-set selection criteria, with median effect size greater than 1 (1.07–1.92) and a cross-validation accuracy of 99% (S1A Fig). A predictivity of 95% was achieved in a validation step on the same discovery MS platform based on a blinded set of triplicate measurements from 6 models (3 responder and 3 non-responder models), including 2 new models that were not part of the discovery set.

The 5-protein response prediction signature was then transferred onto a targeted MS platform to enable quantitative protein measurement from unfractionated lysates. A training set consisting of 9 responder and 17 non-responder xenograft models was used to re-train the classifier based on a multiplexed targeted SRM approach using the 3 best peptides of each of the target proteins as protein quantification reporters (S2 Table). Performance of the signature was then re-assessed on a blinded set of replicate measurements from 6 models (3 responder and 3 non-responder models) that included 2 new models that were not part of the training set. In contrast to the measurements performed using the discovery proteomics platform, only 3 of the 5 proteins part of the signature were robustly quantified within the dynamic range of the SRM platform. PADI and CLIC3 were not detected in all conditions, with the latter protein measured predominantly in the responder models. Initially, re-training of the response prediction signature classifier was achieved by imputing missing values to the median of the respective response group; in a subsequent application of the signature in the validation set, missing values were imputed to the average of the 2 group medians (responders and non-responders), to treat the samples in a blinded fashion. In parallel, we also considered a simplified combined measure, merely summing all 5 markers’ relative protein concentration (using a weight of -1 for DPYSL2 to account for the opposite effect direction for this marker compared to the 4 remaining proteins) to segregate responders from non-responders. Such a universal measure would enable the assessment of the response prediction signature in the ultimate clinical setting in the absence of a training dataset. Both the re-trained SVM predictor and the simplified combined measure demonstrated high selectivity (predictive positive value: 91%) and showed the same trend and separation if analyzed using RNA sequencing or DNA microarray methods, indicating that genomic, transcriptional, and translational information agreed but with the targeted mass spectrometric approach demonstrating overall best performance (S1B Fig). Importantly, based on the data gained in the discovery proteomics validation phase, the performance of a 4- or a 3-protein response prediction signature (without CLIC3 and/or PADI3) was comparable to the original signature if the remaining features were robustly measured (S1C Fig).

### Development and qualification of a 5-plex SRM assay in FFPE tissue

The objective of this study consisted in translating a discovery, 5-plex SRM assay developed in fresh frozen xenograft tumor into a qualified assay for analyzing FFPE primary human tumors collected in a phase Ib clinical trial. While the mass spectrometric process is *per se* not tissue- or peptide-dependent, prevalence of the proteins of interest in the target tumor tissue needed to be determined to ensure the feasibility of the approach. Also, the selection of the SRM reporter peptides required careful review as they might not perform equally well in fresh frozen and formalin-fixed tissue sources [21]. Finally, the algorithm behind the response prediction signature needed to be re-assessed in the formalin-fixed xenografts to confirm the performance in the new setting. Thus, assay development and qualification were divided in 3 stages, each of them with a go/no-go decision point to limit time and expenses in case of failure (Table 1).

**Table 1:** Assay development and qualification plan for the 5-protein signature in FFPE breast cancer tissue.

Stages	Description	Deliverables
**I:** Assay development and prevalence analysis	• Identification of unique peptides amenable to SRM analysis in FFPE tissue and prevalence analysis in selected primary target tissue.	• Selection of suitable reporter peptides for the 5 target proteins (DPYSL2, OAT, CLIC3, GM2A, PADI3) in FFPE matrix. • Prevalence analysis in 20 FFPE tissue samples (10 sqNSCLC^#^ and 10 breast cancer). • GO/NO-GO milestone: detection of 4 out of 5 proteins in 80% of tissue samples (sqNSCLC and/or breast cancer)
**II:** Responder/Non-Responder signature assessment in FFPE matrix	• Selection of the best 3–5 reporter peptides for each protein based on stage I results including at least one peptide previously identified in the SRM assay developed in fresh frozen material. • Establish calibration curves in surrogate matrix. • Target protein measurement in mirrored fresh-frozen/FFPE responder/non-responder xenograft tumor tissue.	• Relative protein abundance in FFPE and fresh-frozen xenografts. • GO/NO- GO milestone: reproduction of prediction protein signature in FFPE xenografts with PPV>80%
**III:** Assay qualification in target tissue	• Selection of best 1–3 reporter peptide(s) for each target protein. • Establish linearity of calibration curves and limit of detection/quantitation in target tumor tissue.	• Establishment of assay linearity, LOD and LLOQ. • GO/NO-GO milestone: the abundance of the target proteins is higher than the assay’s LLOQ based on the results obtained in stages I and II.

^#^sqNSCLC: squamous Non Small Cell Lung Cancer

The first stage of the assay development consisted in selecting the SRM reporter peptides to be measured in the FFPE matrix and in determining the candidate protein’s prevalence in the target tumor tissue. Potential reporter peptides (proteotypic, no methionine, no miss-cleavage, 8 to 20 amino acids in length) were selected from an *in silico* digest of the 5 proteins and transition information was converted to a prototype SRM assay and confirmed by analyzing the recombinant protein digest by LC-MS. Each protein’s best 5 to 9 detected peptides were then assessed in 17 cell lines (various lineages) treated with formalin and in 10 Non Small Cell lung cancer and 10 breast cancer FFPE tissue samples obtained from commercial sources (S4 Table). While several peptides for each of the 5 proteins were identified in lung cancer, CLIC3 and PADI3 were essentially undetectable in breast cancer, indicating lower prevalence in this tissue. However, the non-detectability of these two proteins might also have been due to the absence of an internal standard as a higher signal-to-noise cutoff was used to ensure robust detection. Based on these results, a collection of stable isotope-labelled peptides was ordered (Table 2); the selection included one to two peptides originally used to measure the signature in the fresh frozen xenograft tissues to confirm translatability to the FFPE clinical material. Of note, some peptides could only be detected in primary tissue samples and not in cell lines, demonstrating the importance of developing SRM assays in the appropriate target tissue.

**Table 2:** SRM reporting peptides assessed in the 5-protein signature in cell lines and in breast and lung tissue.

Protein Name	Peptide Sequence	Found in cell lines?	% of positive in Non Small Cell lung cancer	% of positive in breast cancer
**OAT**	LGIILR$	Yes	100%	100%
	FAPPLVIK*$	Yes	100%	100%
	LPSDVVTAVR*$	Yes	100%	100%
	TVQGPPTSDDIFER	Yes	80%	90%
**DPYSL2**	QQAPPVR$	Yes	100%	100%
	VFNLYPR*$	Yes	100%	100%
	SSAEVIAQAR	Yes	100%	100%
	TVTPASSAK*$	Yes	100%	30%
**PADI3**	ILIGGNLPGSSGR$	Yes	60%	0%
	DLINYNK$	No	50%	0%
	VSYEVPR*$	No	40%	0%
	DFLHAQK	No	40%	0%
	TISINQVLSNK*	No	40%	0%
**GM2A**	VDLVLEK*$	Yes	100%	90%
	IESVLSSSGK*$	No	100%	90%
	EGTYSLPK$	No	100%	80%
	EVAGLWIK	No	90%	0%
**CLIC3**	QAPIPAELR$	Yes	80%	20%
	APLEHELAGEPQLR$	Yes	80%	10%
	FLDGDR	Yes	60%	20%
	GVPFTLTTVDTR*	Yes	0%	0%
	DFAPGSQLPILLYDSDAK*	Yes	0%	0%

*: reported peptide included in the SRM assay developed for the fresh frozen xenograft models

$: peptides included in the qualified SRM assay in FFPE tissue.

Shaded cells highlight conditions where a given peptide was not detected.

**Fig 2:**
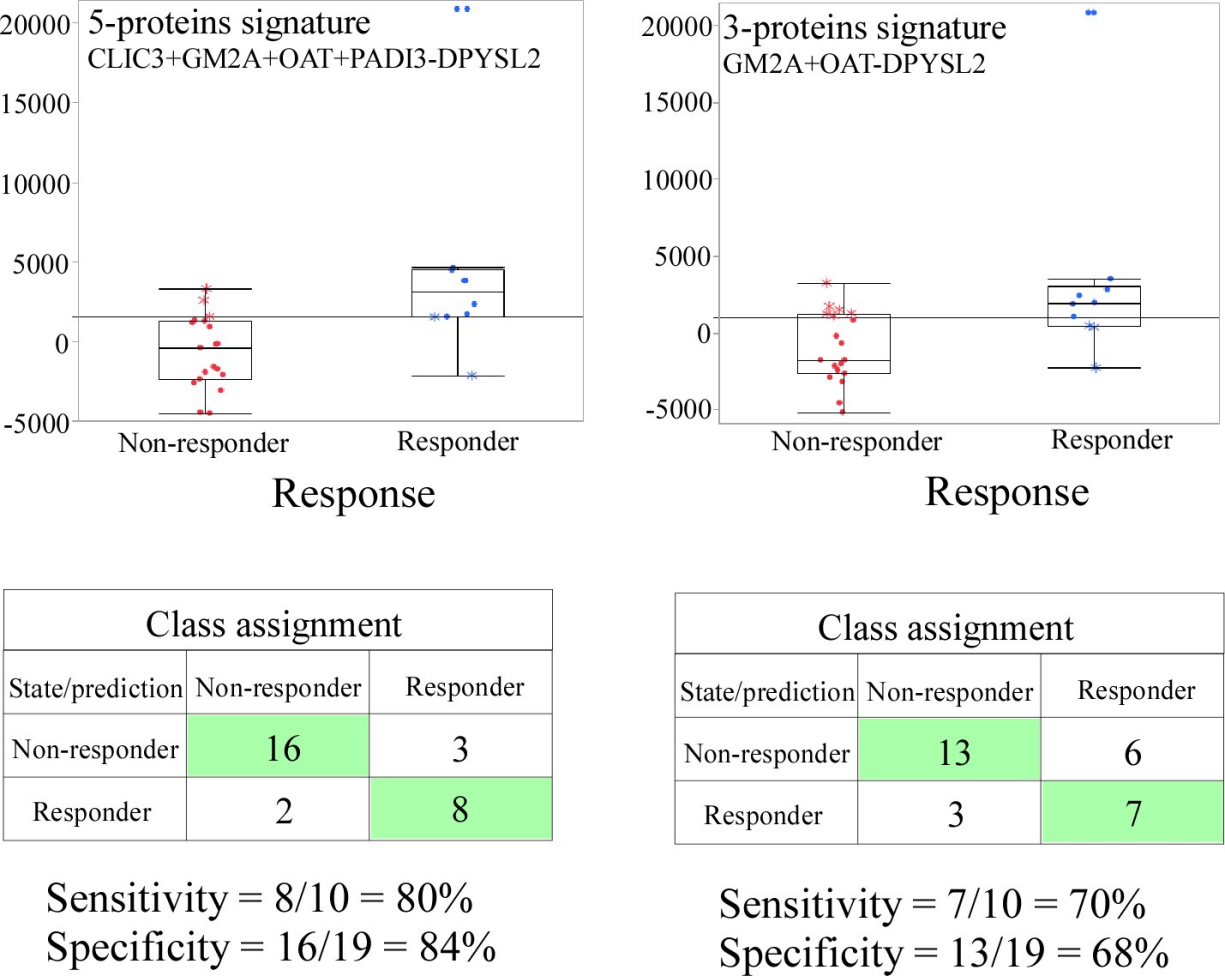
Box plot representations and performance of the 5- and 3-protein signatures predictive for lumretuzumab activity in responder and non-responder FFPE xenograft models. The box plots data distribution includes minimum, first quartile, median, third quartile, and maximum.

The second step of the assay development focused on demonstrating that the response prediction signature was independent of the matrix and could be reproduced in the FFPE xenografts. For this purpose, 10 responder and 19 non-responder formalin-fixed xenograft samples (identical to the original fresh frozen material except for an additional formalin fixation step) were selected to confirm the response prediction algorithm. Three sections of 10 μm thickness were cut from each block and the tumor area was isolated using laser-capture microdissection. The relative levels of the 5 proteins were measured against the spiked internal stable isotope labelled peptides using the prototype SRM assay. Similary to previous results in fresh frozen tumors, the 5 proteins were not consistently detected in all samples (S5 Table). However, missing values were mostly observed in samples where lower amounts of the target proteins were expected, likely due to the constrained sensitivity of the SRM assay (informative missing values) in unfractionated lysates compared to the strategy used in the Discovery Proteomics phase. Therefore, non-detected peptides were imputed using a randomly chosen concentration close to (but below) LLOQ (estimated to lie around 100 amol peptide/μg lysate) to simulate the situation in the tissue. The validation of the response prediction in FFPE tissue is displayed in Fig 2 (the detailed impact of the imputation method on proteins’ abundance distribution is plotted in S2 Fig). The 5-protein response prediction signature in FFPE xenografts showed a sensitivity of 80% and a specificity of 84%, similar albeit slightly lower to what was observed in fresh frozen xenografts, with two false positive samples very close to the signature threshold. Remarkably, the general trends in protein abundance changes were kept despite change of assay format and matrix. The assay performance was poorer if only the three most abundant proteins (DPYSL2, GM2A and OAT, for which there was no missing value) were taken into account. However, it demonstrated that the imputation method did not distort the response prediction signature.

The final step of the assay development and qualification was to qualify and demonstrate the clinical utility of the assay in representative samples. To this effect, the three best performing peptides (respectively two for CLIC3, as determined in the previous development stage of the assay) of each protein were qualified with respect to linearity and lower quantification limits in a *P*. *furiosus* lysate spanning a calibration range from 25 amol to 25 fmol peptide injected in 1 μg total lysate (S3 Fig). For most peptides, the lower limit of quantification (defined as the calibration level above limit of detection whereas CV and recovery were within 20% error) was around 100–150 amol peptide detected per μg lysate injected. The assay’s utility was demonstrated by measuring 18 of the clinical samples and two of the xenograft samples used during initial assay development (S6 Table). The re-measured data was in excellent agreement with the quantification results reported previously in the study. In particular, trends in protein abundance were kept when quantification was performed with the appropriate stable isotope standards instead of using the EGFR peptide as universal calibrator. Also, the use of specific internal peptide standards resulted in lower LLOQs as the cutoff for signal decay was improved by the presence of the standards’ corresponding transitions; this was particularly obvious for the measurement of PADI3 in the breast cancer samples. Overall, these observations provided confidence that the SRM method was of clinical utility to be used in tissue samples obtained from patients.

### Analysis of the putative response prediction signature in patient tumor biopsies from a clinical trial

A total of 32 individual breast cancer tumor biopsies (out of 35 patients enrolled in the clinical trial BP27852 [16]) were submitted for analysis. FFPE blocks were sectioned at 10 μm thickness and H&E stained sections placed on glass slides were evaluated by the in-house pathologist to direct LCM dissection of the tumors. Sufficient material was obtained from 17 patients to assess the response prediction signature by MS while there was insufficient tumor tissue available from the remaining 15 patients. The patient clinical profile and response to treatment is provided in S7 Table. All tissue samples were analyzed in triplicate except for three patients for whom there was not sufficient protein extracted, so that they were evaluated either in single or duplicate analyses. The detailed analysis results are reported in S8 Table.

**Fig 3:**
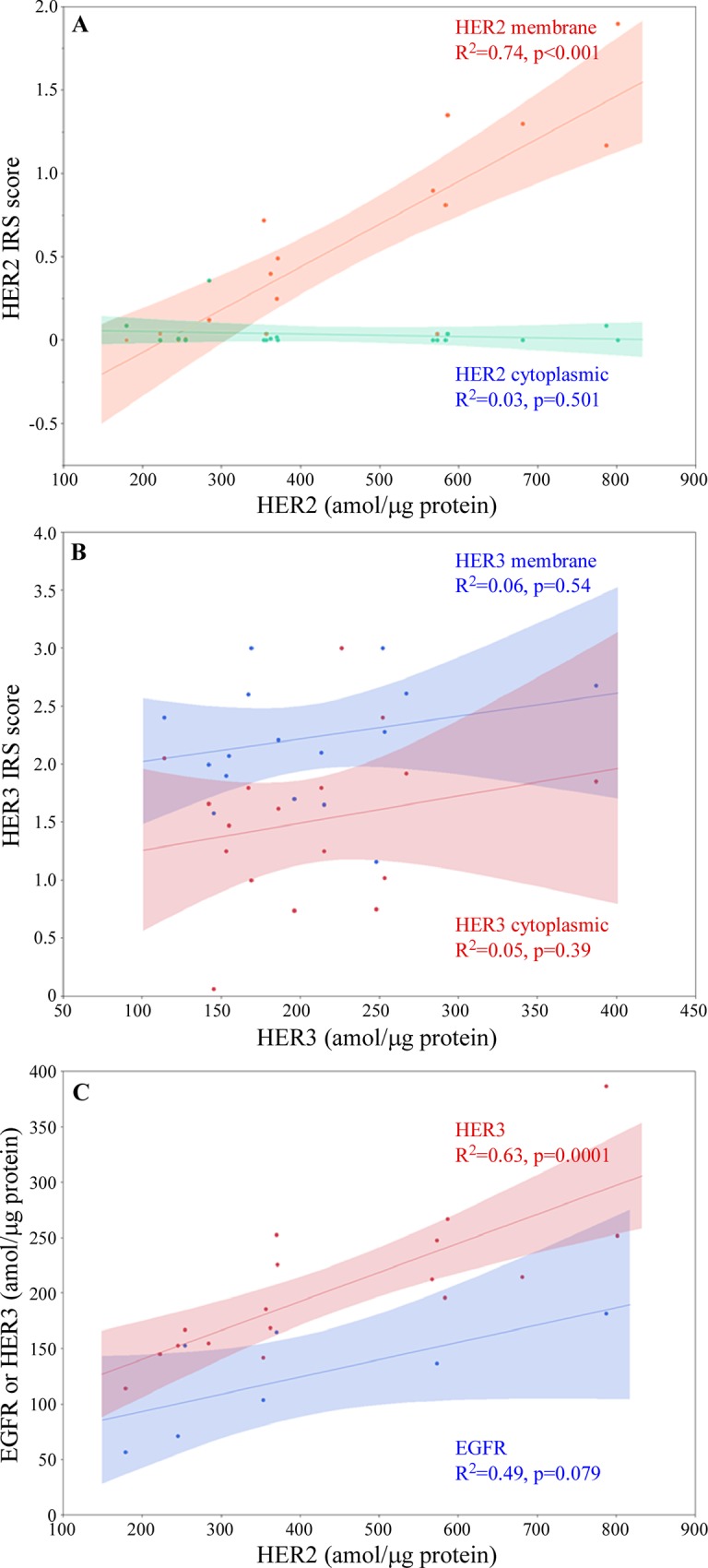
Correlation plots. (A) HER2 IRS scores measured by IHC and HER2 protein concentration measured by SRM (B) HER3 IRS scores measured by IHC and HER3 protein concentration measured by SRM (C) EGFR and HER3 versus HER2 protein concentration measured by SRM. The shaded area represents the 10% confidence interval of the linear fit.

The suitability of the collected samples set for a MS-based approach was assessed by measuring the expression level of EGFR, HER2, and HER3 using established SRM assays as described previously [20]. The results were compared to IHC measurements performed on the same samples (Fig 3). Reportable EGFR levels were found in only 5 of the 17 investigated samples (EGFR amounts below LLOQ were detected in two additional samples), in good agreement with the low signal for this protein measured by IHC. HER2 and HER3 levels were measurable in all samples. In particular, total HER2 levels as assessed by SRM correlated linearly with the HER2 membranous immune reactivity score (IRS) determined by IHC (Fig 3A), in concordance with previously published data [22]. Reported levels for total HER2 by SRM were indicative for non- or weakly over-expressed HER2 protein, which is consistent with the “HER2-low” eligibility criteria used to enroll patients in this clinical trial. In contrast, there was no correlation between HER3 IHC data and reported total protein levels by SRM (Fig 3B). Total HER3 expression level in non-cancerous tissue is believed to be low, possibly below the LLOQ of this assay. The fact that all investigated tumors contained significant levels of HER3 indicates that this protein was likely over-expressed in the tumor (in concordance with the “HER3-high” eligibility criteria used to enroll patients in the trial). The apparent lack of correlation between IHC and SRM may be due to the lack of dynamic range of the ultrasensitive HER3 IHC procedure used in this study [11] and the difficulty to quantitatively assess protein levels using this approach. Interestingly, using SRM as a reporting method, HER3 and possibly EGFR levels appear to linearly correlate with HER2 levels (Fig 3C), possibly indicative for the presence of HER2 heterodimers in the tumor.

The 5 proteins included in the response prediction signature were measured concomitantly in the SRM assay performed for each patient’s sample. Similar to what was observed in the assay development stages, DPYSL2, OAT and GM2A were measured and quantified in all samples while PADI and CLIC3 were most of the time below the assay’s sensitivity threshold. Consistency between peptides for each given protein was excellent (median %CV = 9.4), with a correlation between peptide concentrations that was always ≥ 0.9, indicating that the reported peptide levels were an appropriate surrogate for global protein concentration in a sample.

**Fig 4:**
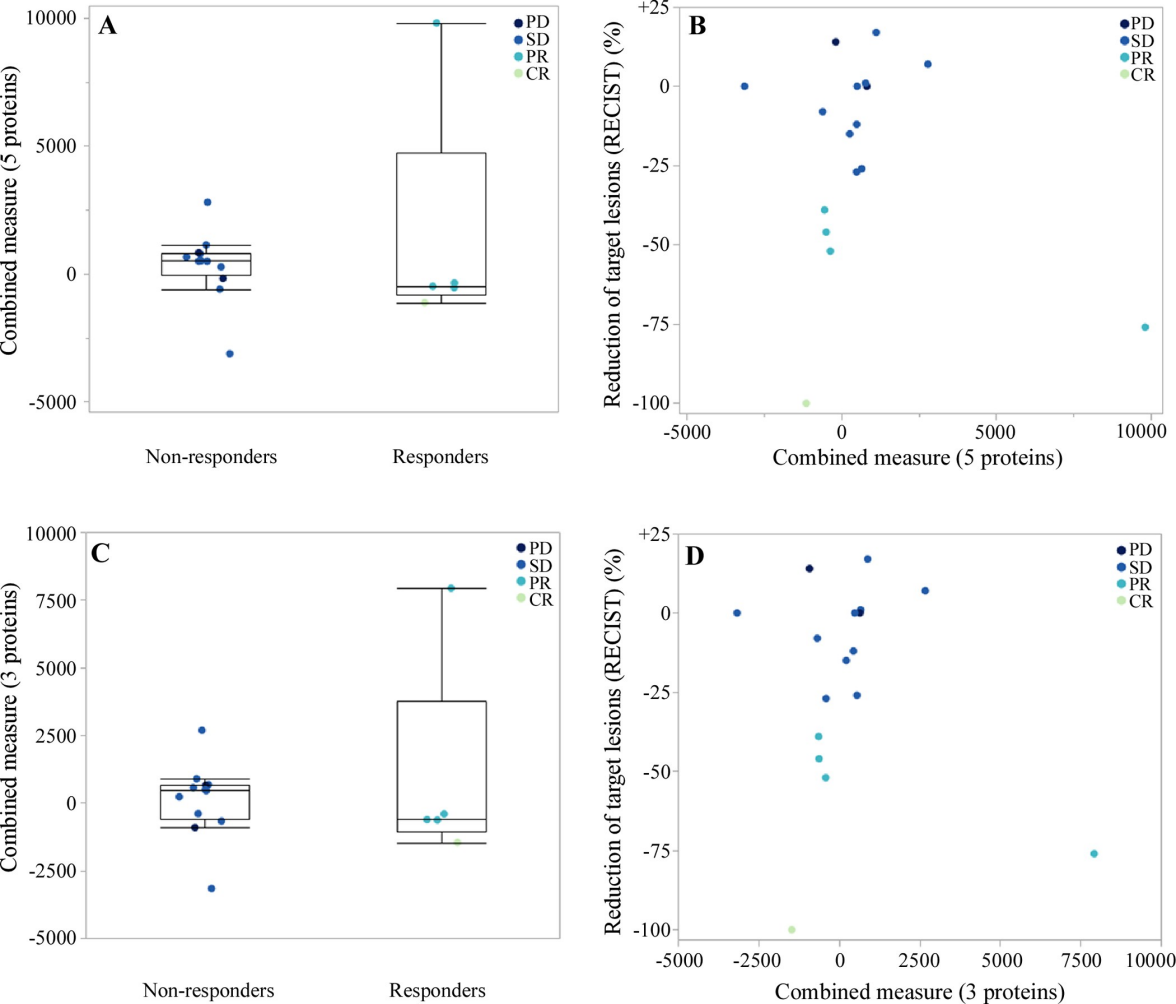
Box plot representations and correlation of the 5- and 3-protein signatures with patient response. (A, C): Box plot representations of the 5-protein (A) and the 3-protein (C) signature separated by patient response; the box plots data distribution includes minimum, first quartile, median, third quartile, and maximum. (B, D): Correlation of the 5-protein (B) and the 3-protein (D) signature to the individual patient response assessed by RECIST; the signatures’ combined measure is obtained by summing the concentration of the proteins CLIC3+GM2A+OAT+PADI3-DPYSL2 (5-protein signature) or GM2A+OAT-DPYSL2 (3-proteins signature). PD: progressive disease; SD: stable disease; PR: partial response; CR: complete response.

The re-qualified response prediction signature in FFPE tissue was assessed in the donors’ samples and compared to their respective RECIST response status. Similar to what was implemented in the assay development phase, the missing PADI and CLIC3 quantification values were assumed to be informative missing (below LLOQ) and were imputed as described previously. The results for the 5-protein (including the imputed PADI and CLIC3 values) and for the 3-protein (considering only DPYSL2, OAT and GM2A) signatures are shown in Fig 4. Responding and non-responding patients could not be differentiated by the response prediction signature with only one responder patient clearly standing out in the expected effect direction. The clear separation of this single responder was mostly due to very elevated OAT and CLIC3 protein levels compared to all other patients. Unfortunately, the limited number of responders analyzed in the cohort does not allow hypothesizing whether this separation is due to chance or whether this was due to a meaningful biological effect.

## Discussion

In a heterogeneous disease such as breast cancer, histopathology-based factors such as tumor size, grade, nodal status, hormonal and HER2 receptor status are commonly used to guide treatment. However, patients with similar disease characteristics may still experience different outcomes, stressing the need for additional predictive, diagnostic and prognostic biomarkers (reviewed in [23]). In recent years, MS-based proteomics platforms have made significant progresses towards clinical applications, especially in biomarker discovery and verification, where challenges associated with analyses of clinical samples include wide dynamic range of protein concentrations in tissue samples and the need to perform high throughput and accurate quantification of candidate biomarker proteins [24]. A MS-based strategy for measurement of clinical samples provides several advantages over other protein-centric platforms, especially if the candidate biomarkers of interest were initially discovered using a proteomics strategy. In particular, the primary detector, the mass spectrometer, is used in both discovery and assay development and signal generation relies on the same biochemical and biophysical processes, which facilitates method transfer and qualification. Importantly, a multiplexed protein assay is best geared towards a MS-based platform as all signals are processed and qualified in parallel in each sample, whilst time and effort invested in method development and qualification does not significantly increase. Also, assay development and qualification by mass spectrometric-based approaches has been formalized and stringent guidelines have been put in place to ensure reproducibility and performance of the assays put forward [25,26].

In this study, a targeted MS multiplexed approach was facilitated by the initial work conducted in the proteomics discovery step and the following SRM assay build performed in fresh frozen xenografts. Assay development and qualification in FFPE human tissue samples included re-qualification of the reporter peptides, prevalence analysis, qualification of the assay, and confirmation of the response prediction algorithm. We took advantage of the multiplexing capability of the assay including reporter peptides specific for EGFR, HER2 and HER3 to confirm overall suitability of the methodology by comparing the levels of these proteins with the IHC analysis in the same samples. The full assay transfer, development, and qualification was achieved within a 7 months period, a record time compared to other protein-centric technology platform, and suitable performance was demonstrated in the matrix of relevance confirming its classification as a Tier two “fit-for-purpose” assay [26]. The concomitant assessment of EGFR, HER2 and HER3 provided additional evidence that the full workflow (including tissue processing) was appropriate for the measurement of the 5-protein response prediction signature. Seventeen individual clinical samples were processed and analyzed by multiplex SRM. While the levels of HER2 were in line with the results obtained by IHC, levels of HER3 did not match IHC results, possibly due to limitation of dynamic range for the later platform. Further, DPYSL2, OAT, and GM2A were measured and quantified in all samples while PADI and CLIC3 were most of the time below the assay’s sensitivity threshold, similar to that observed in the assay qualification samples. Overall, the combined measure of the target proteins (per the response prediction algorithm) did not match the patient response as assessed by RECIST, irrespective of whether a 3- or a 5-protein combination was used. It is to note, however, that the observable biological variability made it very challenging to assess a multiparametric response signature in a sampling size as few as 17 samples, especially as the validation study demonstrated a weaker performance of the signature in FFPE compared to what was measured in the original fresh frozen setting.

A discrepancy between pre-clinical models and the outcome in this study may be due to several factors, including biological (lineage) differences between xenografts, and primary human tumors. Also, the response signature was geared towards prediction of a monotherapy mode of action, which did not match the clinical situation where lumretuzumab was given in combination with paclitaxel and pertuzumab. Overall, it also can be argued that the confirmation of the response prediction signature (the clinical response of the tumor due to lumretuzumab mode of action) was hampered by the low number of responders observed in the trial, tumor heterogeneity, and by the challenge to translate findings obtained with single agent usage to a combination treatment setting in a clinical trial. However, most importantly, the low number of samples that could be effectively measured in the assay combined with a considerably larger biological heterogeneity than observed in the xenografts impaired an unbiased evaluation of the response signature. Residual material from pre-dose FFPE tumors was not available in sufficient amount due to high demand in tumor tissue for numerous testing or too heterogeneous to be dissected out using laser capture microdissection.

In this study, we have demonstrated one possible avenue on how to enable a large number of biomarkers to be tested in clinical settings and to rapidly assess their intrinsic value. In particular, an innovative and rapid development of a targeted mass spectrometric-based assay to assess a response prediction signature was highly warranted as neither baseline HER2 or HER3 protein expression (as measured by IHC) nor Heregulin mRNA expression (as measured by PCR) were associated with clinical response to lumretuzumab in Breast Cancer [16]. As pointed out in a recent review [27], Wiktorowicz and Brasier deplore that very few biomarker panels have been translated to clinical practice to date despite great interest and potential impact; however, they note that an initial proposal on how to validate biomarkers and demonstrate clinical utility [28] might have been too optimistic (and generic) for this purpose. In parallel to their proposed refined strategy for a more rational design for candidate biomarker development, we would also argue for the technological need for a more rapid and systematic testing campaign of these biomarkers in the clinical setting, such as proposed in this study. In particular, we strongly believe that the rapid switch of technological platforms between discovery and clinical qualification is one of the critical hurdles faced in a biomarker development plan, due to the efforts and costs to develop and qualify immunological reagents against unproven targets. While it is certain that a majority of candidate biomarkers will not withstand clinical evaluation, we do still need a rational process and a path forward to rapidly test and identify the few that stand a chance for further development and validation using finite resources. In this respect, MS-based platforms may provide a cost- and time-effective strategy to achieve an objective evaluation of candidate biomarkers in the clinical setting with the ability to assess their value in comparison to co-measured established biomarkers. It is to note, however, that the successful application of MS-based platforms will critically depend on the availability of an appropriate sample size and quality to enable proper statistics and unbiased evaluation, implying a timely inclusion of the assay in the clinical protocol. In our laboratory, we are currently investigating on how to integrate targeted and un-targeted mass spectrometric-based exploratory strategies to analyze tissue samples collected during clinical trials. We are confident that procedure harmonization and a dedicated sampling strategy will dramatically raise the probability of success of future proteomics studies.

## Supporting information

S1 Fig Evaluation of the 5-features response prediction signature measured by discovery proteomics, targeted mass spectrometry, Affymetryx, or RNAseq. (PPTX)

S2 Fig Determination of protein concentration before and after sample imputation to remove artefactual «zero-values» due to the limited sensitivity of the SRM measurement. (PPTX)

S3 Fig Calibration curves and determination of LOD and LLOQ for each peptide included in the SRM assay developed to measure the 5-protein response signature in FFPE tissue. (PPTX)

S1 Table Cell lines and patient-derived samples used in the present study to build and validate the response prediction signature. (XLSX)

S2 Table Peptide precursor and product ions used for quantitative analysis of target proteins in fresh frozen matrix. (XLSX)

S3 Table Peptide precursor and product ions used for quantitative analysis of target proteins in FFPE matrix. (XLSX)

S4 Table Evaluation of the 5 proteins in-silico selected peptides in FFPE breast and lung cancer tissue samples. (XLSX)

S5 Table Measurement of the 5-proteins signature in responder and non-responder FFPE xenografts. (XLSX)

S6 Table Measurement of the 5-protein response signature in lung and breast cancer FFPE tissue and in two selected xenografts. (XLSX)

S7 Table Summary of patient clinical data enrolled in trial BP27582. (XLSX)

S8 Table Measurement of the 5-protein signature in FFPE tissue samples from patients enrolled in the trial BP27582. (XLSX)
