# Supplementary material for: Translation and evaluation of a pre-clinical 5-protein response prediction signature in a breast cancer phase Ib clinical trial
Source: PLoS One. 2019 Mar 21;14(3):e0213892. doi: 10.1371/journal.pone.0213892 (PMC6428264; doi:10.1371/journal.pone.0213892)
Supplement: S1 Fig — (PPTX) [file pone.0213892.s001.pptx]

## Slide 1
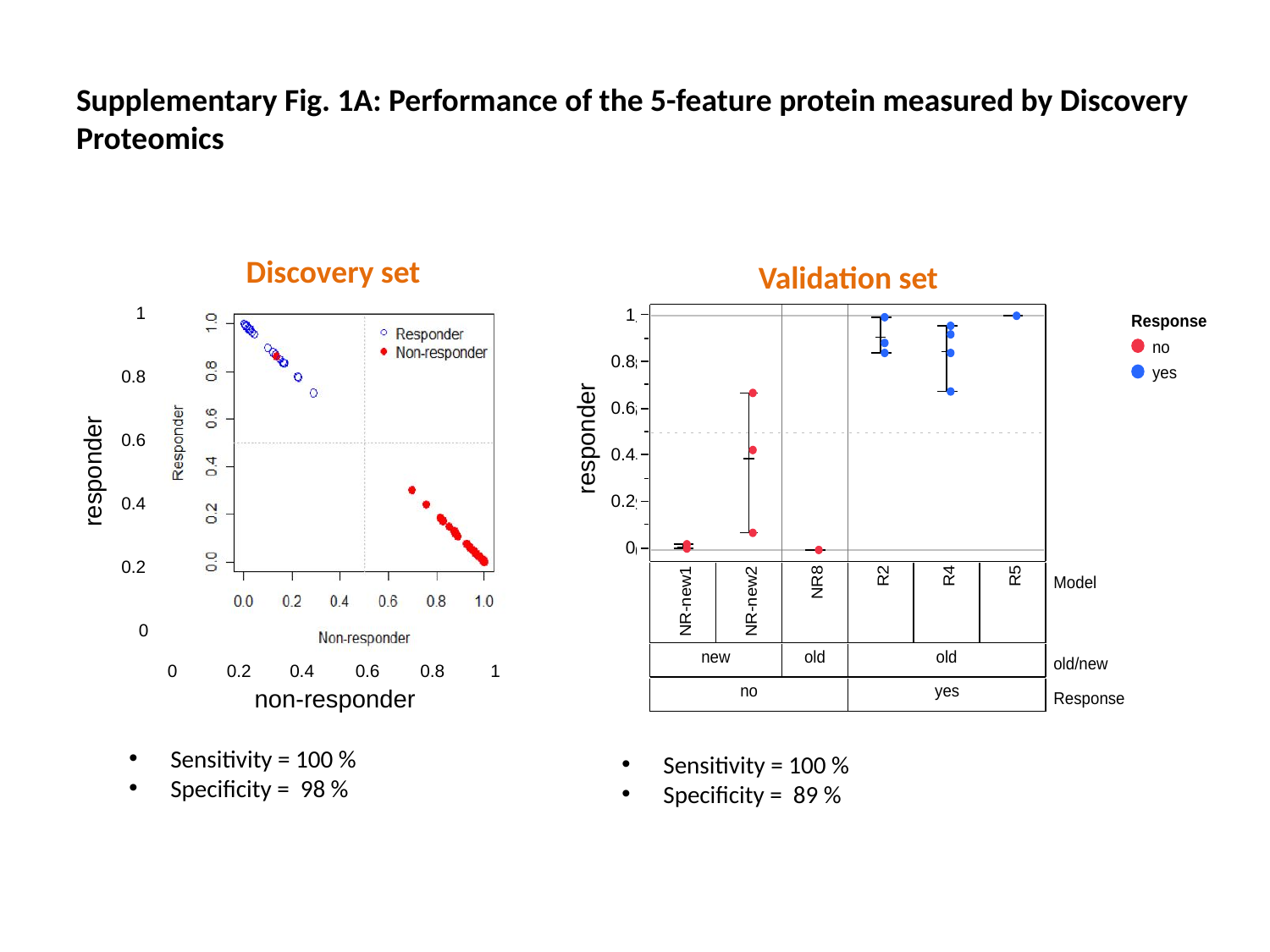

# Supplementary Fig. 1A: Performance of the 5-feature protein measured by Discovery Proteomics
Discovery set
Validation set
1
0.8
0.6
responder
0.4
0.2
0
0
0.2
0.4
0.6
0.8
1
non-responder
1
0.8
0.6
responder
0.4
0.2
0
Sensitivity = 100 %
Specificity = 98 %
Sensitivity = 100 %
Specificity = 89 %

## Slide 2
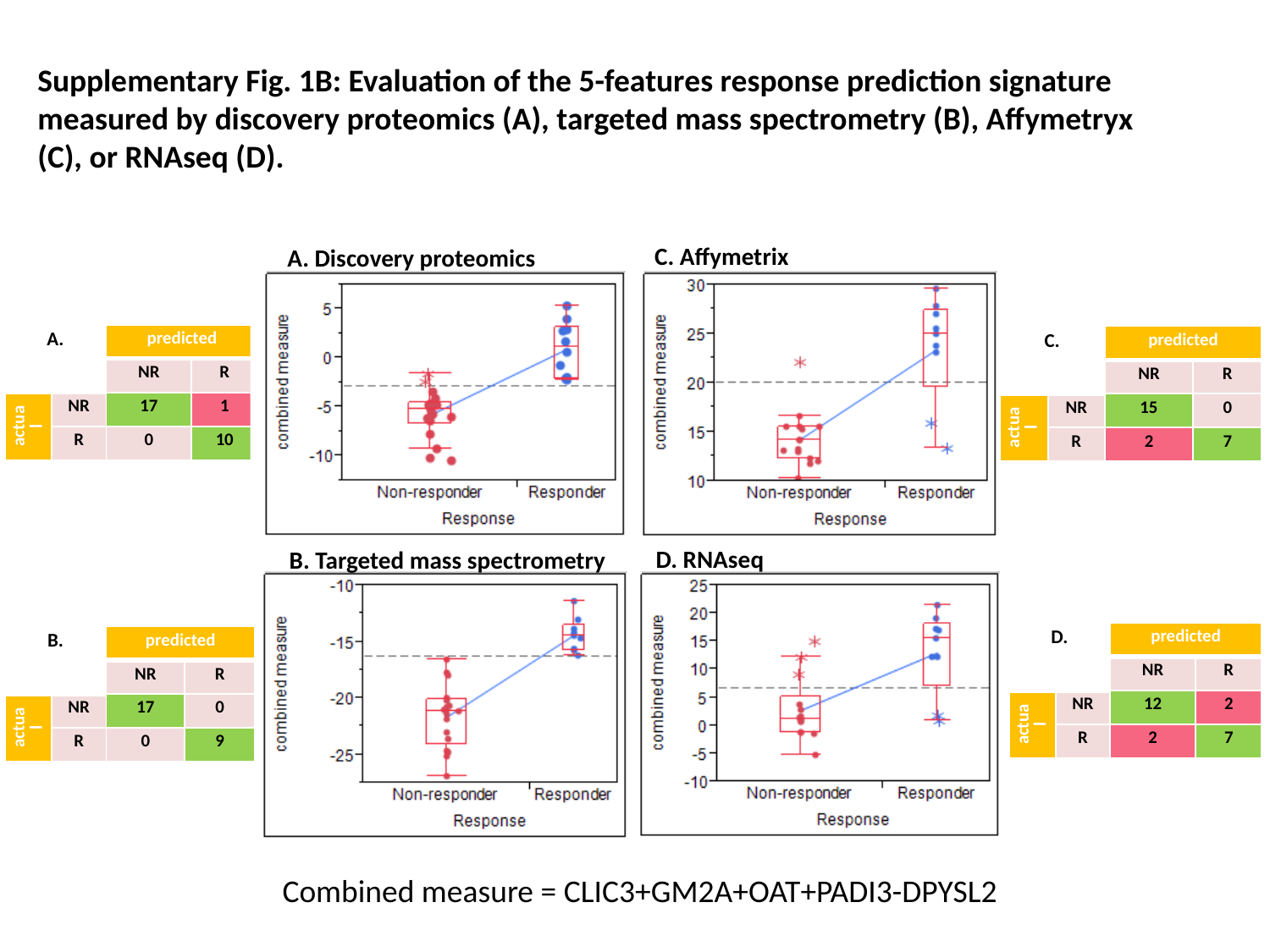

# Supplementary Fig. 1B: Evaluation of the 5-features response prediction signature measured by discovery proteomics (A), targeted mass spectrometry (B), Affymetryx (C), or RNAseq (D).
C. Affymetrix
A. Discovery proteomics
| A. | | predicted | |
| --- | --- | --- | --- |
| | | NR | R |
| actual | NR | 17 | 1 |
| | R | 0 | 10 |
| C. | | predicted | |
| --- | --- | --- | --- |
| | | NR | R |
| actual | NR | 15 | 0 |
| | R | 2 | 7 |
D. RNAseq
B. Targeted mass spectrometry
| D. | | predicted | |
| --- | --- | --- | --- |
| | | NR | R |
| actual | NR | 12 | 2 |
| | R | 2 | 7 |
| B. | | predicted | |
| --- | --- | --- | --- |
| | | NR | R |
| actual | NR | 17 | 0 |
| | R | 0 | 9 |
Combined measure = CLIC3+GM2A+OAT+PADI3-DPYSL2

## Slide 3
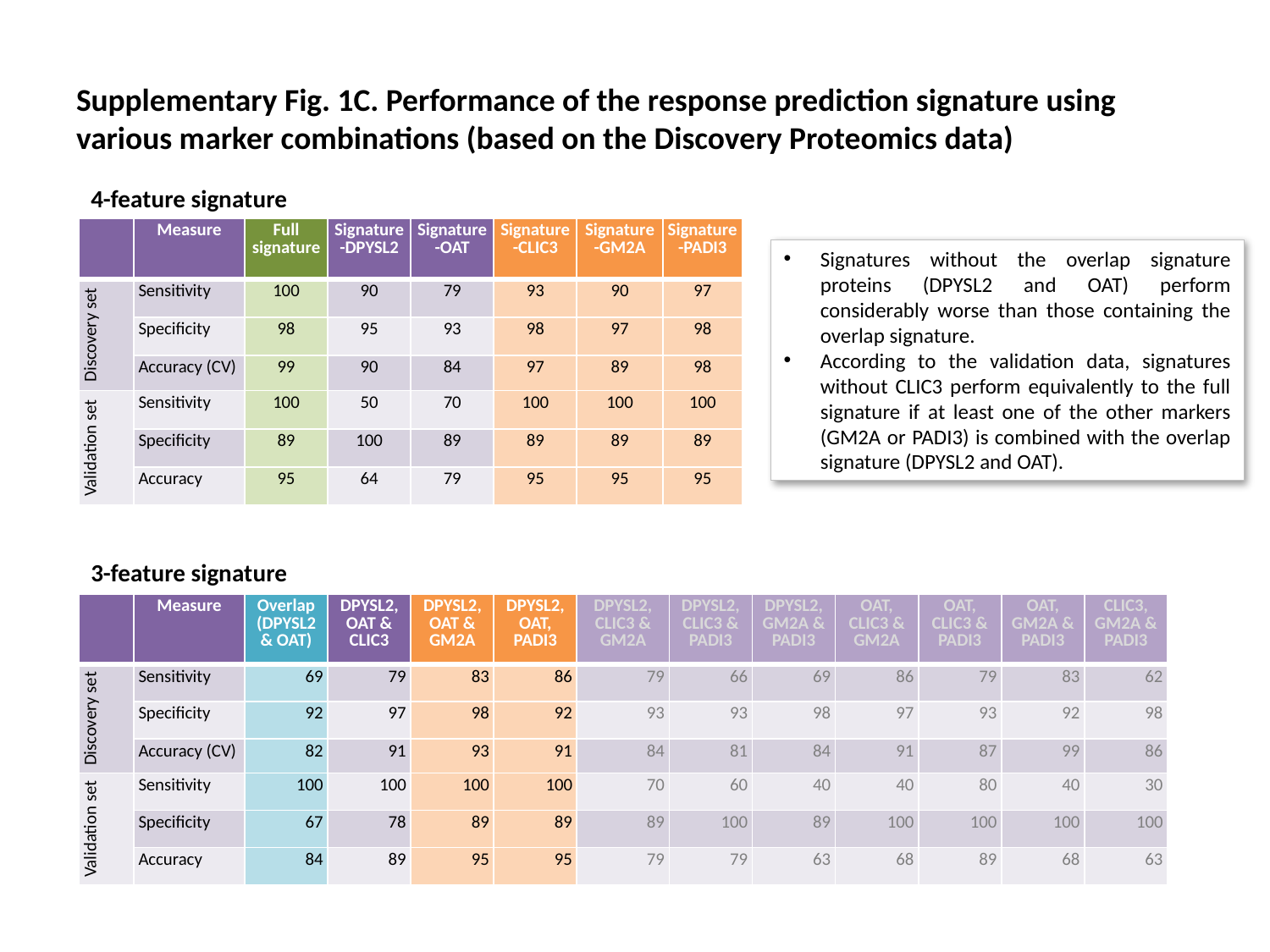

# Supplementary Fig. 1C. Performance of the response prediction signature using various marker combinations (based on the Discovery Proteomics data)
4-feature signature
| | Measure | Full signature | Signature -DPYSL2 | Signature -OAT | Signature -CLIC3 | Signature -GM2A | Signature -PADI3 |
| --- | --- | --- | --- | --- | --- | --- | --- |
| Discovery set | Sensitivity | 100 | 90 | 79 | 93 | 90 | 97 |
| | Specificity | 98 | 95 | 93 | 98 | 97 | 98 |
| | Accuracy (CV) | 99 | 90 | 84 | 97 | 89 | 98 |
| Validation set | Sensitivity | 100 | 50 | 70 | 100 | 100 | 100 |
| | Specificity | 89 | 100 | 89 | 89 | 89 | 89 |
| | Accuracy | 95 | 64 | 79 | 95 | 95 | 95 |
Signatures without the overlap signature proteins (DPYSL2 and OAT) perform considerably worse than those containing the overlap signature.
According to the validation data, signatures without CLIC3 perform equivalently to the full signature if at least one of the other markers (GM2A or PADI3) is combined with the overlap signature (DPYSL2 and OAT).
3-feature signature
| | Measure | Overlap (DPYSL2 & OAT) | DPYSL2, OAT & CLIC3 | DPYSL2, OAT & GM2A | DPYSL2, OAT, PADI3 | DPYSL2, CLIC3 & GM2A | DPYSL2, CLIC3 & PADI3 | DPYSL2, GM2A & PADI3 | OAT, CLIC3 & GM2A | OAT, CLIC3 & PADI3 | OAT, GM2A & PADI3 | CLIC3, GM2A & PADI3 |
| --- | --- | --- | --- | --- | --- | --- | --- | --- | --- | --- | --- | --- |
| Discovery set | Sensitivity | 69 | 79 | 83 | 86 | 79 | 66 | 69 | 86 | 79 | 83 | 62 |
| | Specificity | 92 | 97 | 98 | 92 | 93 | 93 | 98 | 97 | 93 | 92 | 98 |
| | Accuracy (CV) | 82 | 91 | 93 | 91 | 84 | 81 | 84 | 91 | 87 | 99 | 86 |
| Validation set | Sensitivity | 100 | 100 | 100 | 100 | 70 | 60 | 40 | 40 | 80 | 40 | 30 |
| | Specificity | 67 | 78 | 89 | 89 | 89 | 100 | 89 | 100 | 100 | 100 | 100 |
| | Accuracy | 84 | 89 | 95 | 95 | 79 | 79 | 63 | 68 | 89 | 68 | 63 |
