# Supplementary material for: Translation and evaluation of a pre-clinical 5-protein response prediction signature in a breast cancer phase Ib clinical trial
Source: PLoS One. 2019 Mar 21;14(3):e0213892. doi: 10.1371/journal.pone.0213892 (PMC6428264; doi:10.1371/journal.pone.0213892)
Supplement: S2 Fig — (PPTX) [file pone.0213892.s002.pptx]

## Slide 1
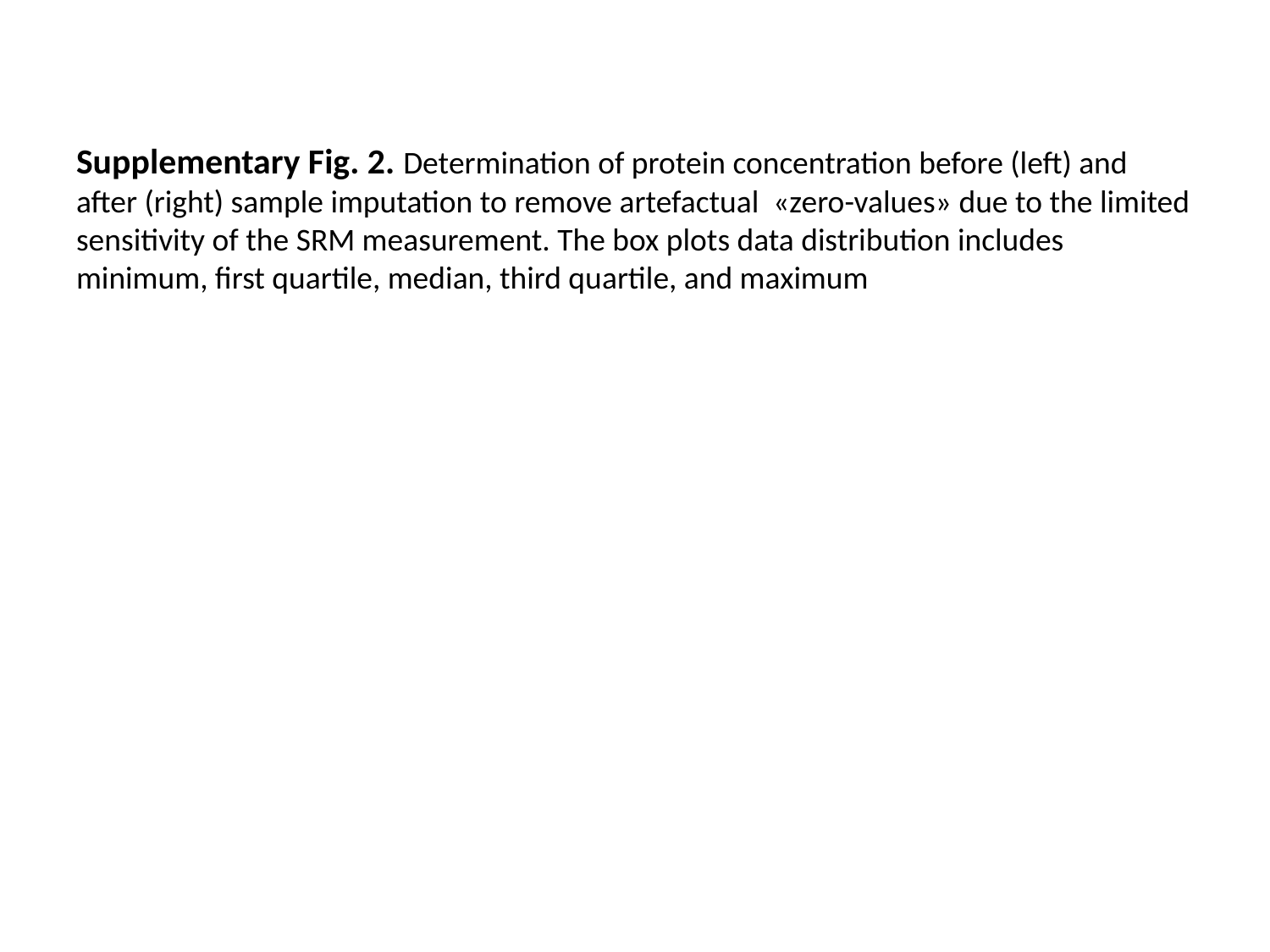

# Supplementary Fig. 2. Determination of protein concentration before (left) and after (right) sample imputation to remove artefactual «zero-values» due to the limited sensitivity of the SRM measurement. The box plots data distribution includes minimum, first quartile, median, third quartile, and maximum

## Slide 2
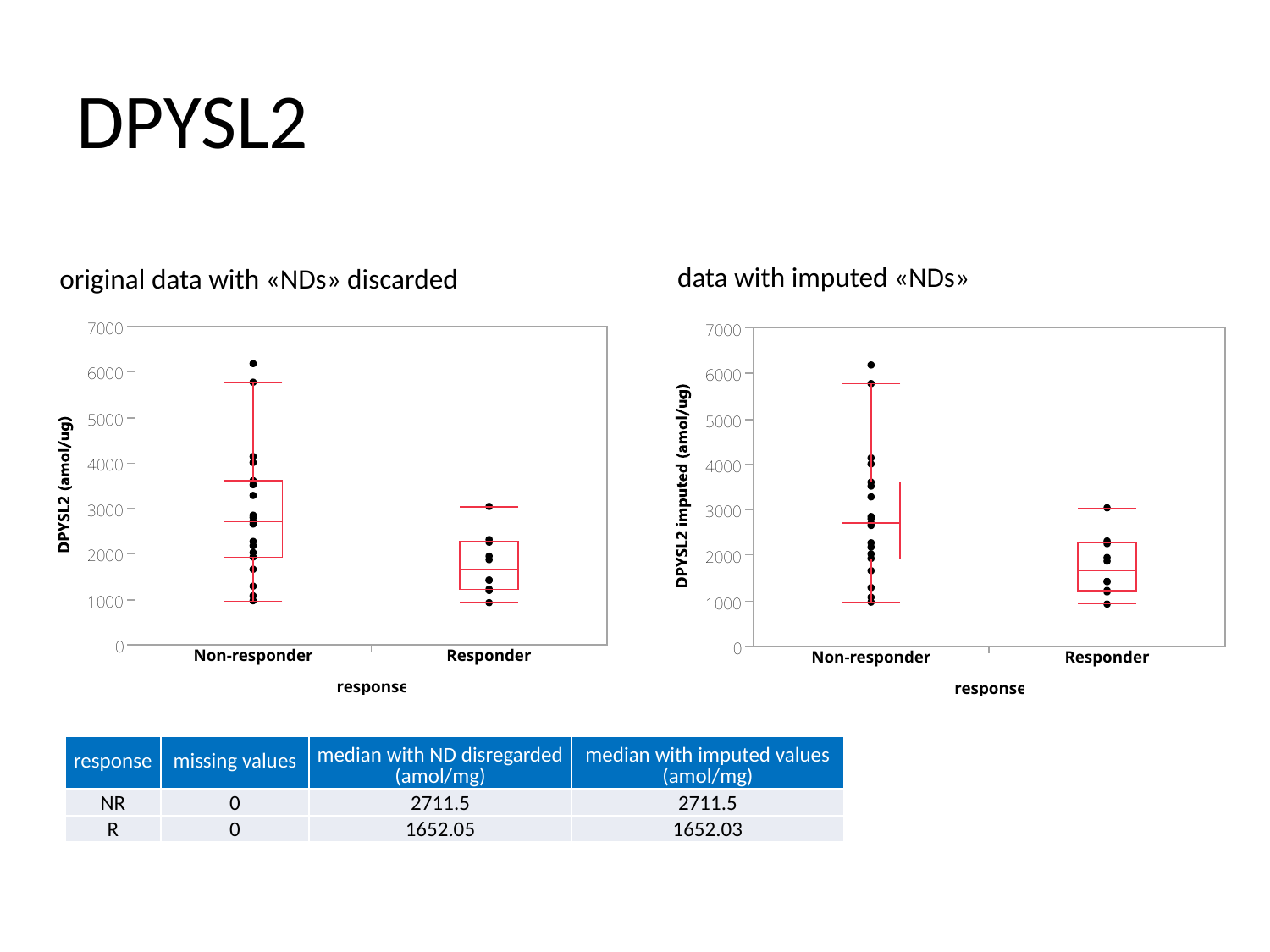

# DPYSL2
data with imputed «NDs»
original data with «NDs» discarded
| response | missing values | median with ND disregarded (amol/mg) | median with imputed values (amol/mg) |
| --- | --- | --- | --- |
| NR | 0 | 2711.5 | 2711.5 |
| R | 0 | 1652.05 | 1652.03 |

## Slide 3
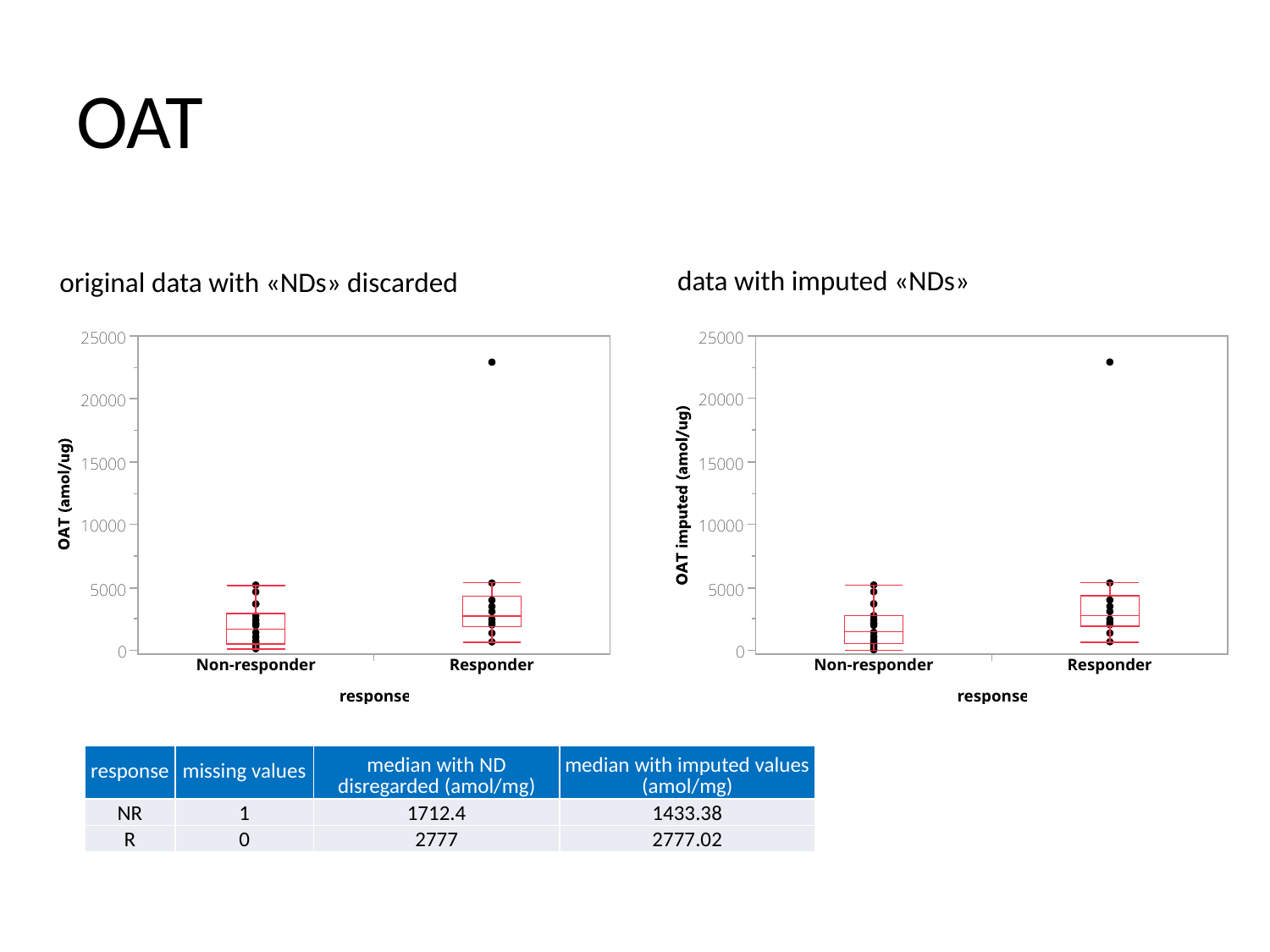

# OAT
data with imputed «NDs»
original data with «NDs» discarded
| response | missing values | median with ND disregarded (amol/mg) | median with imputed values (amol/mg) |
| --- | --- | --- | --- |
| NR | 1 | 1712.4 | 1433.38 |
| R | 0 | 2777 | 2777.02 |

## Slide 4
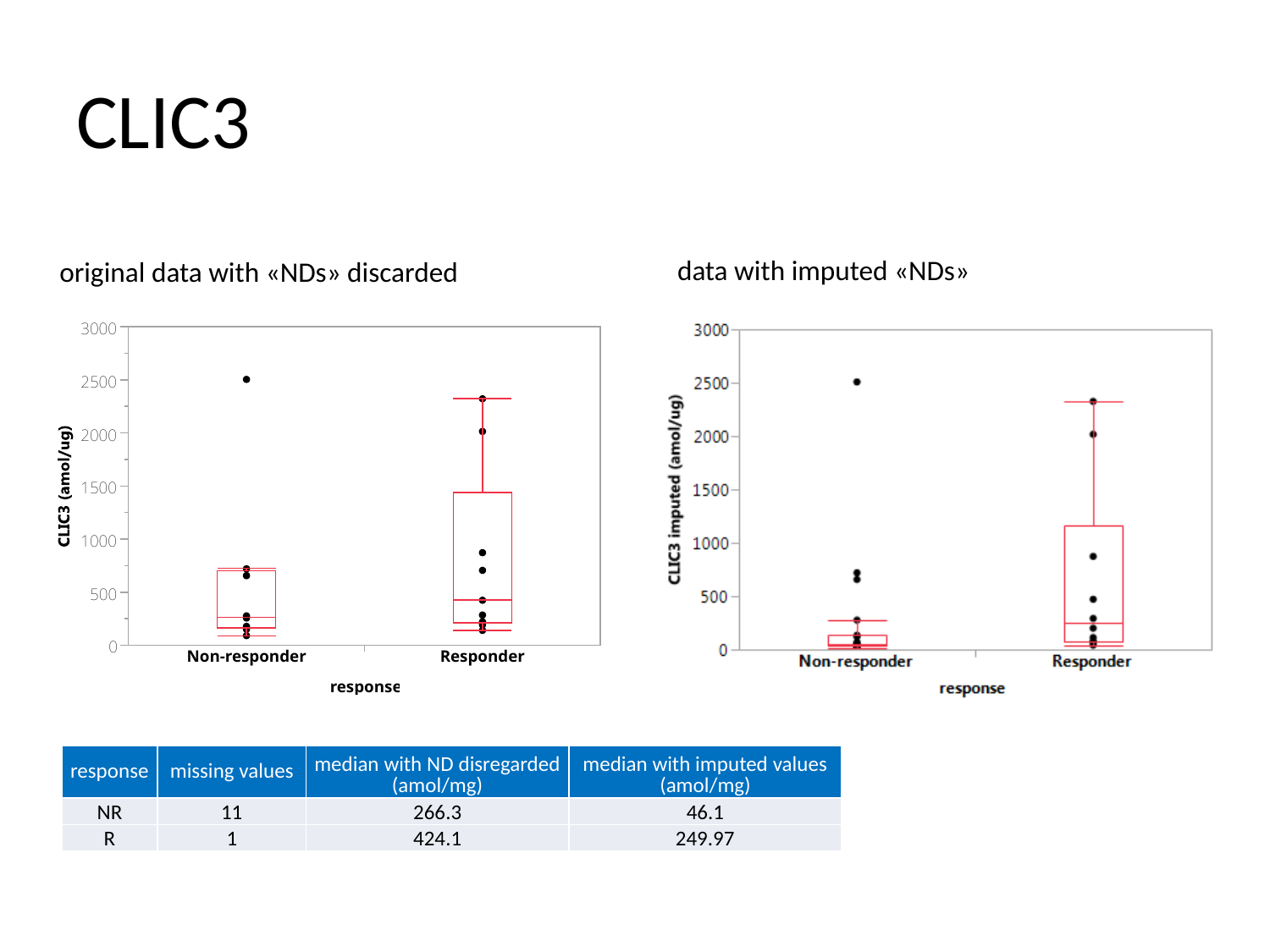

# CLIC3
data with imputed «NDs»
original data with «NDs» discarded
| response | missing values | median with ND disregarded (amol/mg) | median with imputed values (amol/mg) |
| --- | --- | --- | --- |
| NR | 11 | 266.3 | 46.1 |
| R | 1 | 424.1 | 249.97 |

## Slide 5
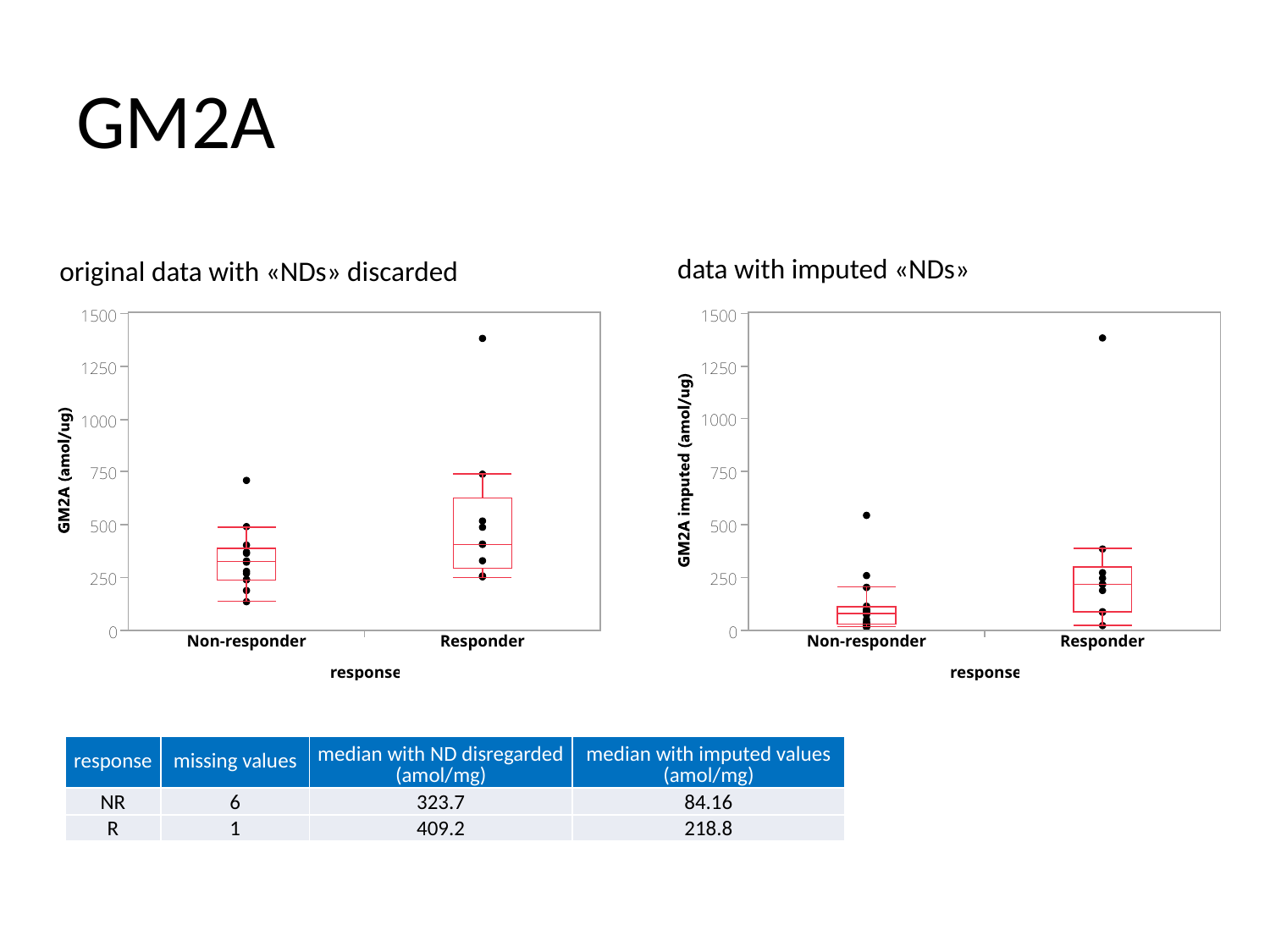

# GM2A
data with imputed «NDs»
original data with «NDs» discarded
| response | missing values | median with ND disregarded (amol/mg) | median with imputed values (amol/mg) |
| --- | --- | --- | --- |
| NR | 6 | 323.7 | 84.16 |
| R | 1 | 409.2 | 218.8 |

## Slide 6
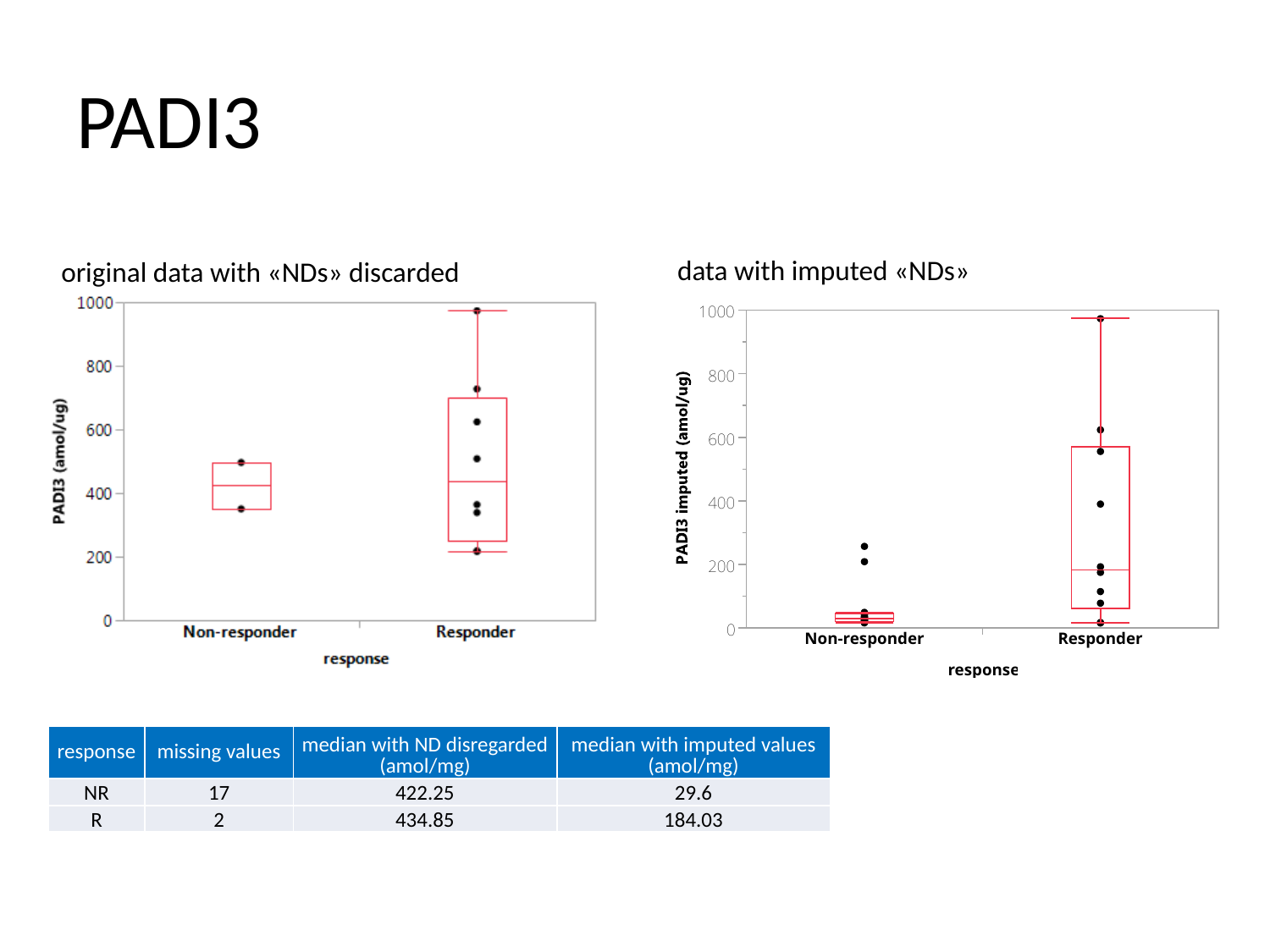

# PADI3
data with imputed «NDs»
original data with «NDs» discarded
| response | missing values | median with ND disregarded (amol/mg) | median with imputed values (amol/mg) |
| --- | --- | --- | --- |
| NR | 17 | 422.25 | 29.6 |
| R | 2 | 434.85 | 184.03 |
