# Supplementary material for: Translation and evaluation of a pre-clinical 5-protein response prediction signature in a breast cancer phase Ib clinical trial
Source: PLoS One. 2019 Mar 21;14(3):e0213892. doi: 10.1371/journal.pone.0213892 (PMC6428264; doi:10.1371/journal.pone.0213892)
Supplement: S3 Fig — (PPTX) [file pone.0213892.s003.pptx]

## Slide 1
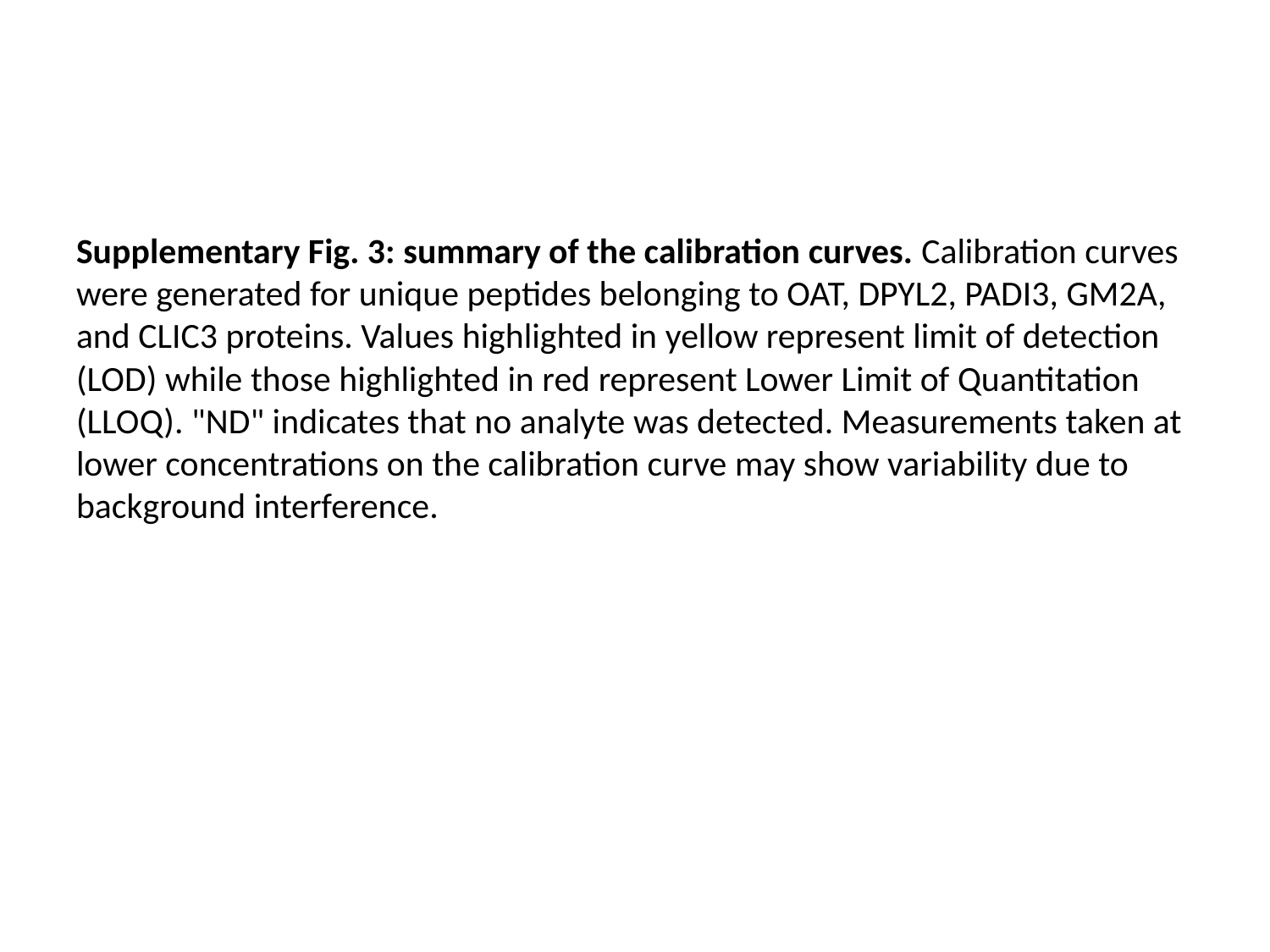

Supplementary Fig. 3: summary of the calibration curves. Calibration curves were generated for unique peptides belonging to OAT, DPYL2, PADI3, GM2A, and CLIC3 proteins. Values highlighted in yellow represent limit of detection (LOD) while those highlighted in red represent Lower Limit of Quantitation (LLOQ). "ND" indicates that no analyte was detected. Measurements taken at lower concentrations on the calibration curve may show variability due to background interference.

## Slide 2
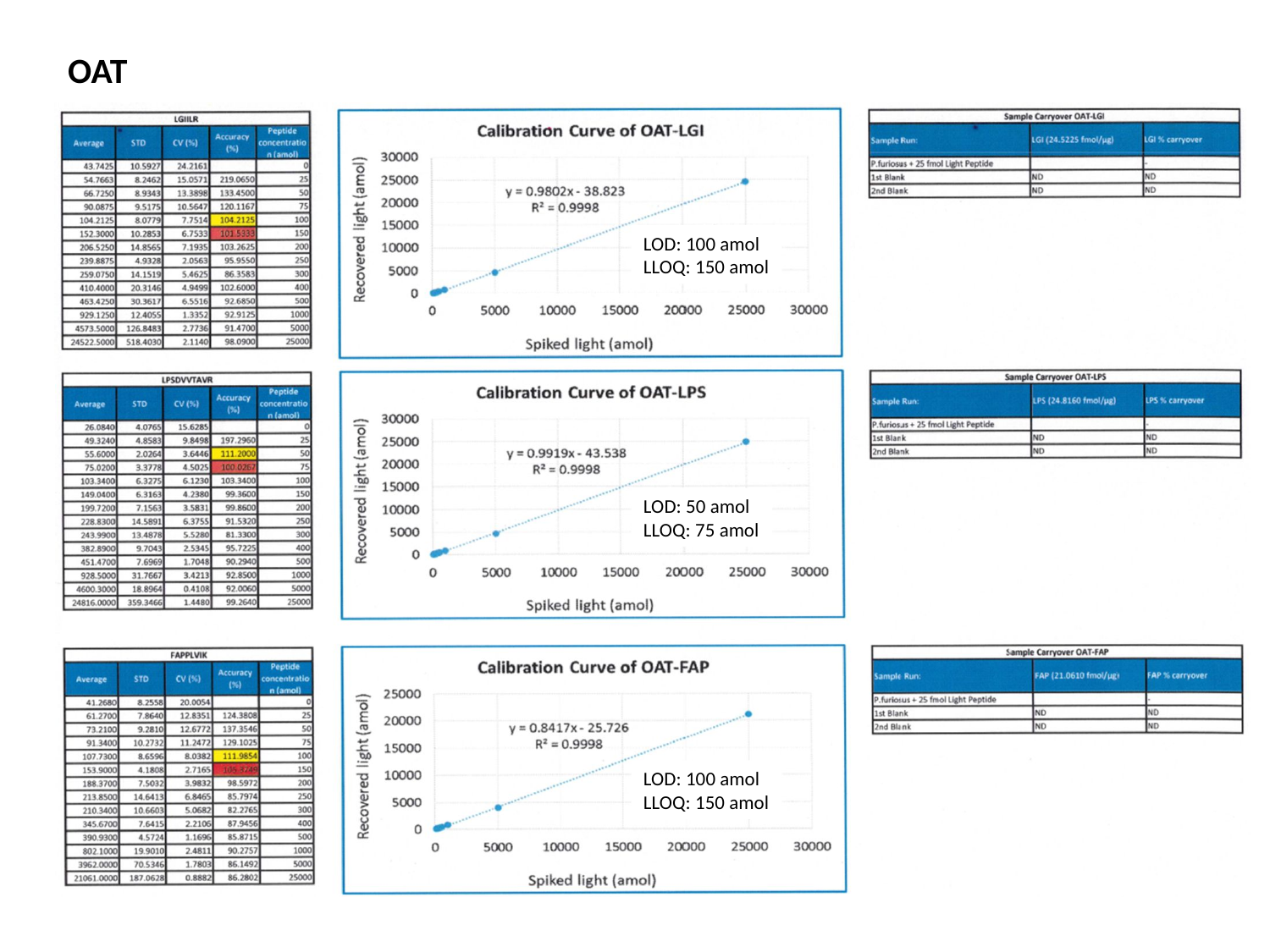

OAT
LOD: 100 amol
LLOQ: 150 amol
LOD: 50 amol
LLOQ: 75 amol
LOD: 100 amol
LLOQ: 150 amol

## Slide 3
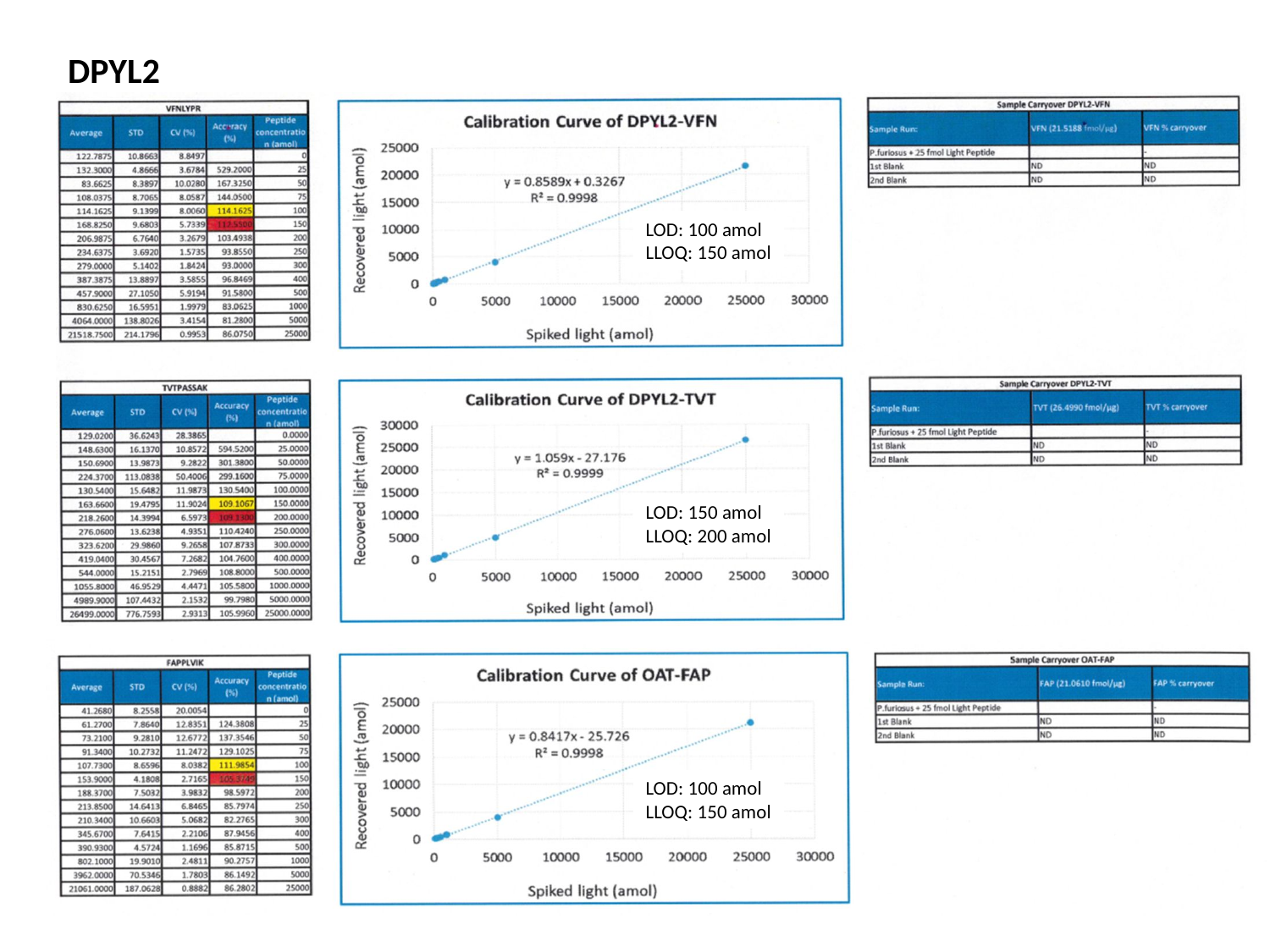

DPYL2
LOD: 100 amol
LLOQ: 150 amol
LOD: 150 amol
LLOQ: 200 amol
LOD: 100 amol
LLOQ: 150 amol

## Slide 4
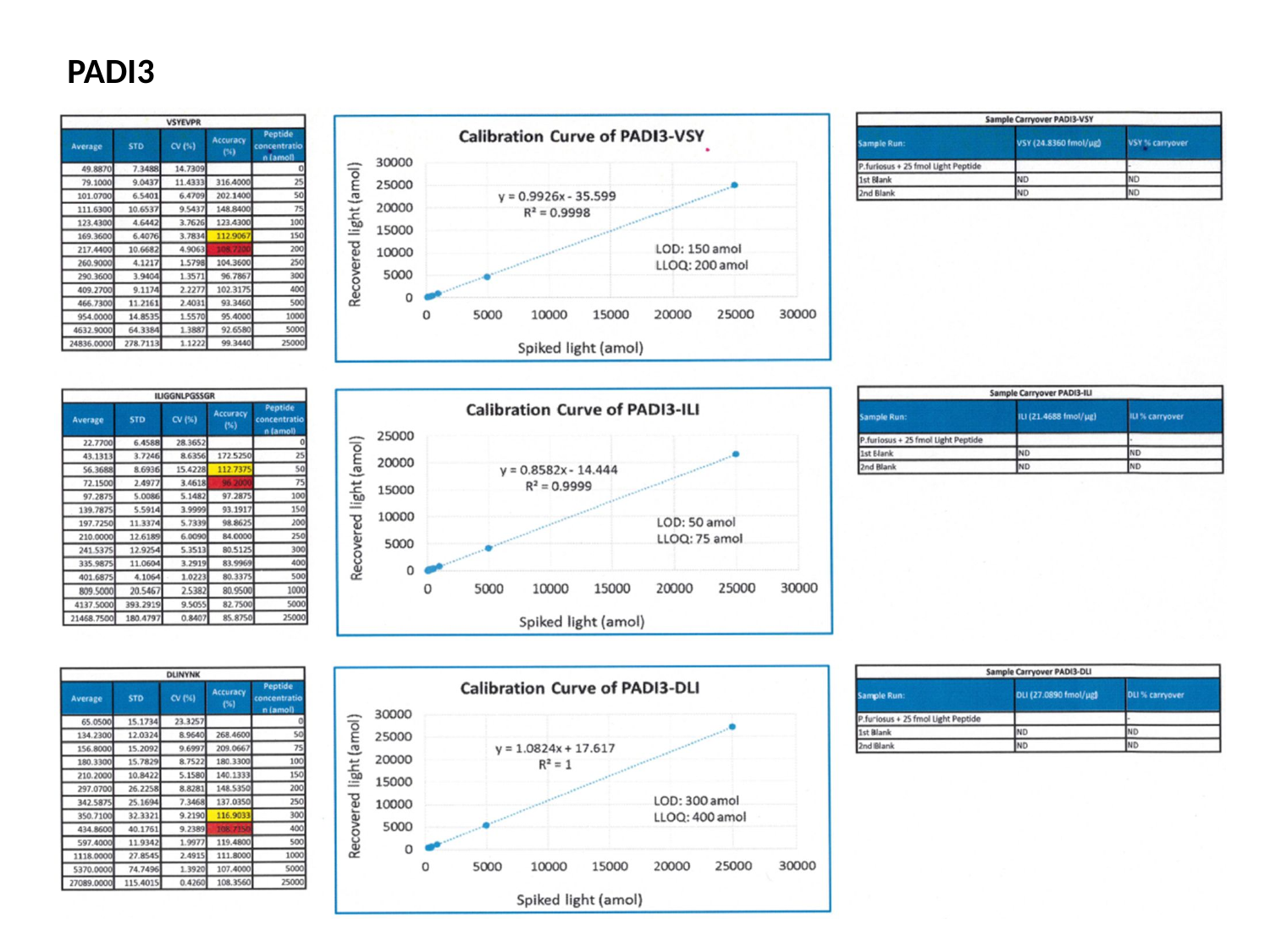

PADI3

## Slide 5
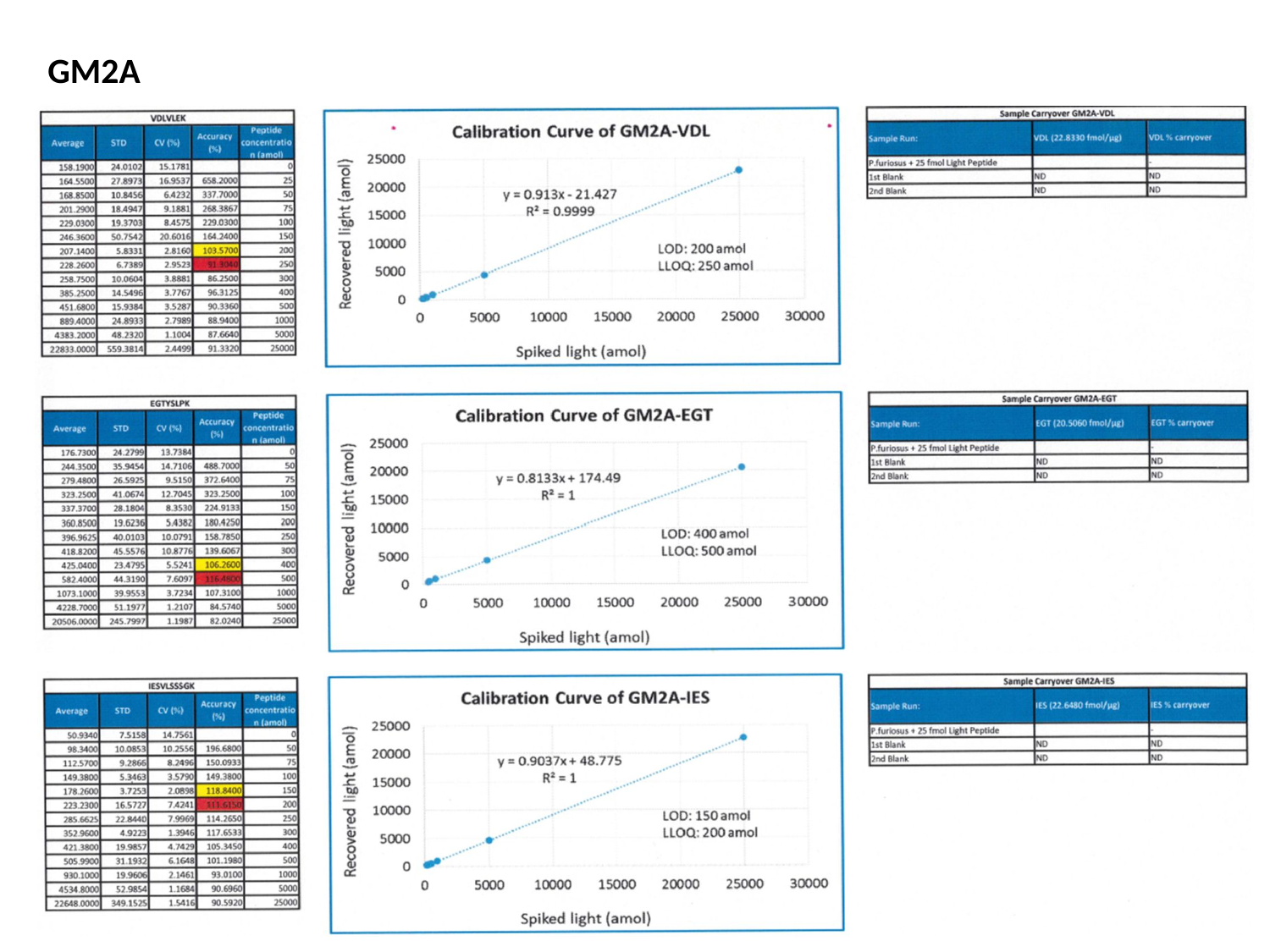

GM2A

## Slide 6
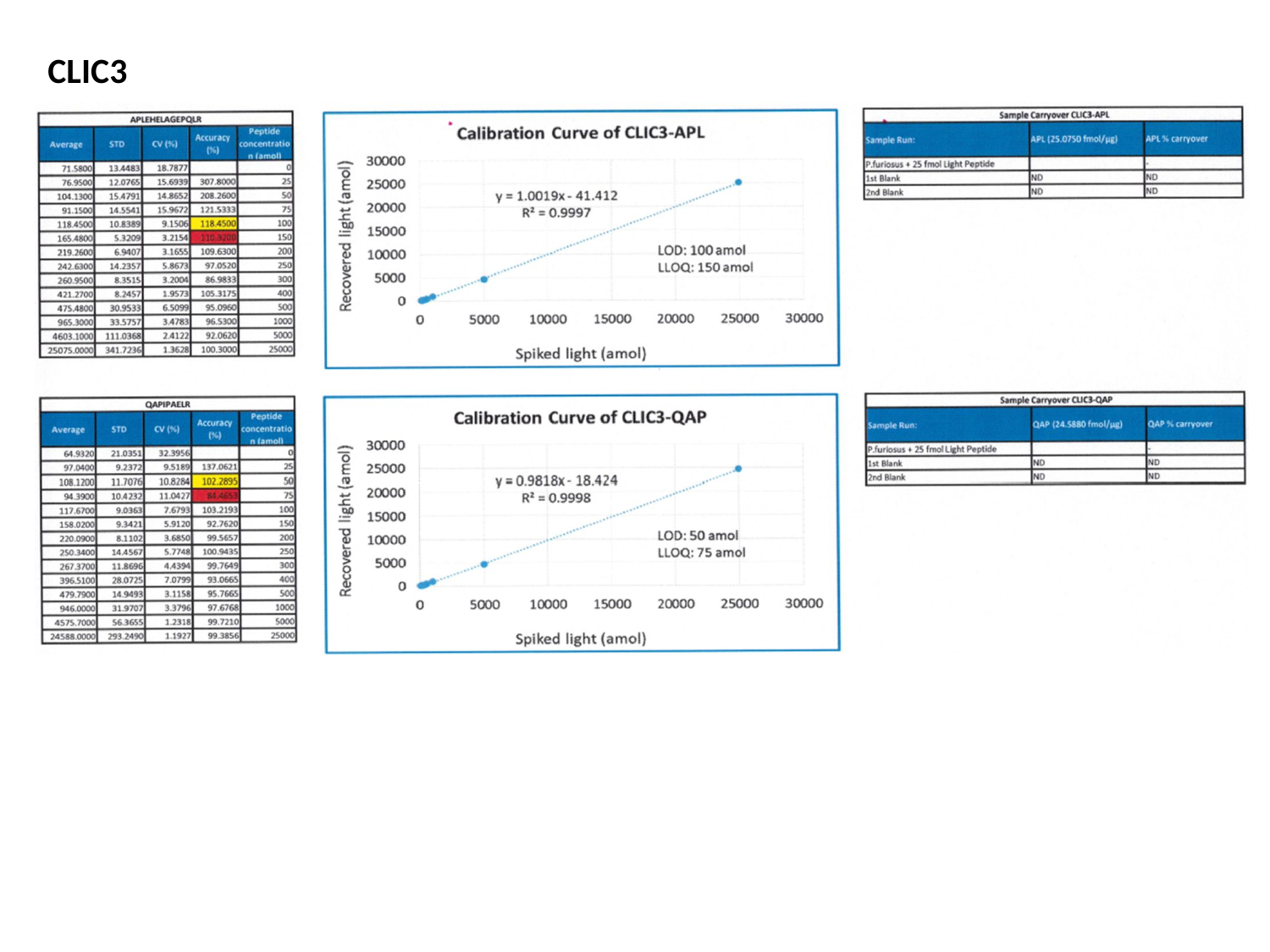

CLIC3
